# Supplementary material for: Heterophilic and homophilic cadherin interactions in intestinal intermicrovillar links are species dependent
Source: PLoS Biol. 2021 Dec 6;19(12):e3001463. doi: 10.1371/journal.pbio.3001463 (PMC8691648; doi:10.1371/journal.pbio.3001463)
Supplement: S1 Fig — Multiple sequence alignments comparing each EC repeat and MAD10 of PCDH24 from 13 different species. Each alignment is colored by percent identity, with white being the lowest percent identity and dark blue being the highest. Sites of N-linked (blue in human, green in mouse) and O-linked (purple in human, red in mouse) glycosylation are denoted by a colored circle. An asterisk (*) indicates sites Y67 and Y71 mutated in binding assays. Secondary structure elements observed in the crystal structures of hs PCDH24 EC1-2 and mm PCDH24 EC1-3 are illustrated below the respective repeats. Residues at the antiparallel homophilic hs PCDH24 EC1-2 II interface (>30% buried surface area) are denoted by a light blue bar. Calcium-binding motifs are indicated above the sequences, which are numbered according to the human protein. Species are abbreviated as follows: Homo sapiens (Hs), Mus musculus (Mm), Sus scrofa (Ss), Gallus gallus (Gg), Aptenodytes forsteri (Af), Parus major (Pm), Anolis carolinensis (Ac), Crocodylus porosus (Cp), Thamnophis elegans (Te), Danio rerio (Dr), Oryzias melastigma (Om), Mastacembelus armatus (Ma), and Xenopus tropicalis (Xt). Species were chosen based on sequence availability, coverage of entire extracellular domain, and taxonomical diversity. Accession numbers and species can be found in S2 Table. EC, extracellular cadherin; PCDH24, protocadherin-24. (PDF) [file pbio.3001463.s001.pdf]

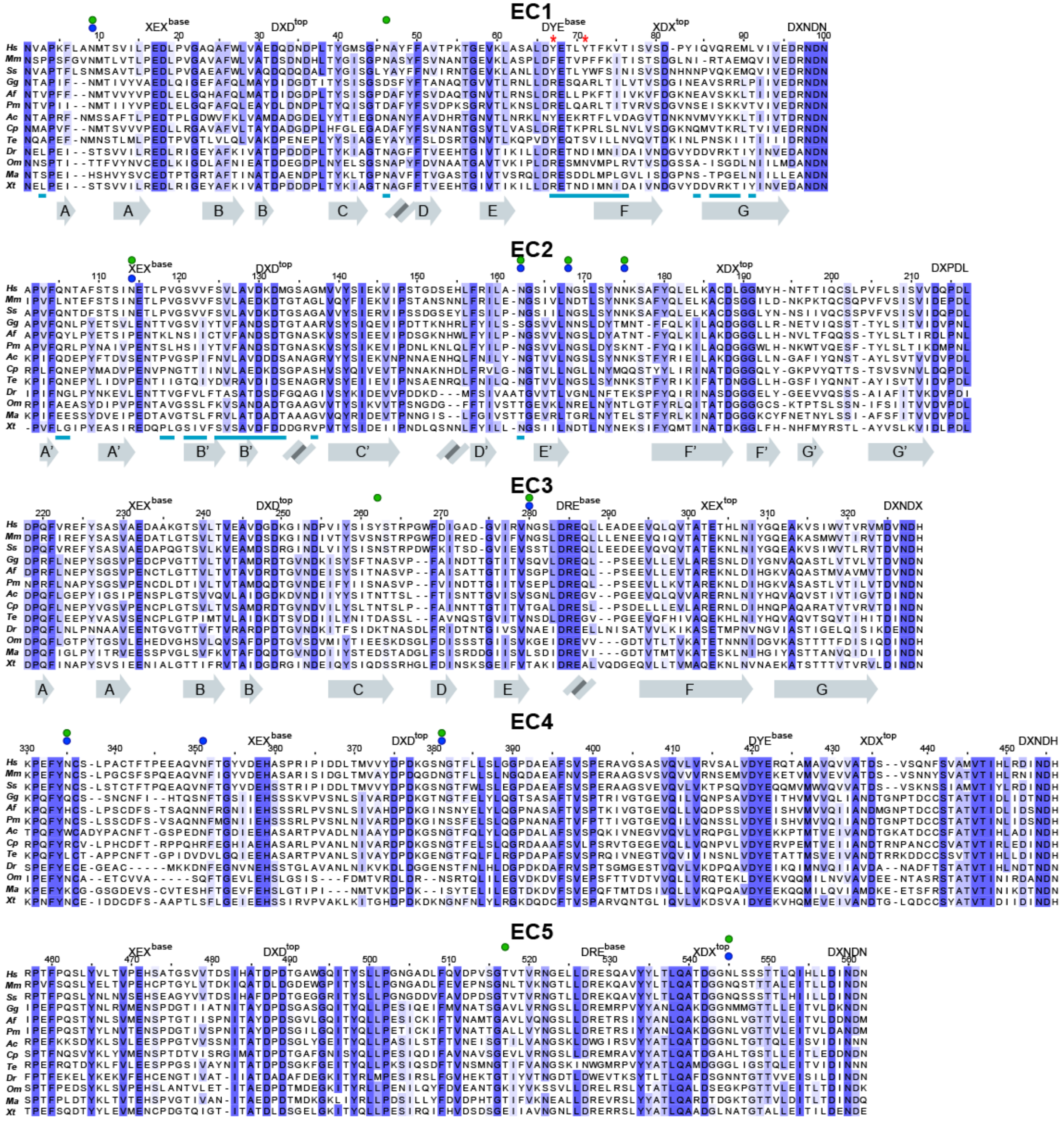
EC1
XEX base
DXD top
DYE base
XDX top
DXNDN
Hs
Mm
Ss
Gg
Af
Pm
Ac
Cp
Te
Dr
Om
Ma
Xt
A
A
B
B
C
D
E
F
G
EC2
XEX base
DXD top
XDX top
DXPDL
A'
A'
B'
B'
C'
D'
E'
F'
F'
G'
G'
EC3
XEX base
DXD top
DRE base
XEX top
DXNDX
A
A
B
B
C
D
E
F
G
EC4
XEX base
DXD top
DYE base
XDX top
DXNDH
EC5
XEX base
DXD top
DRE base
XDX top
DXNDN

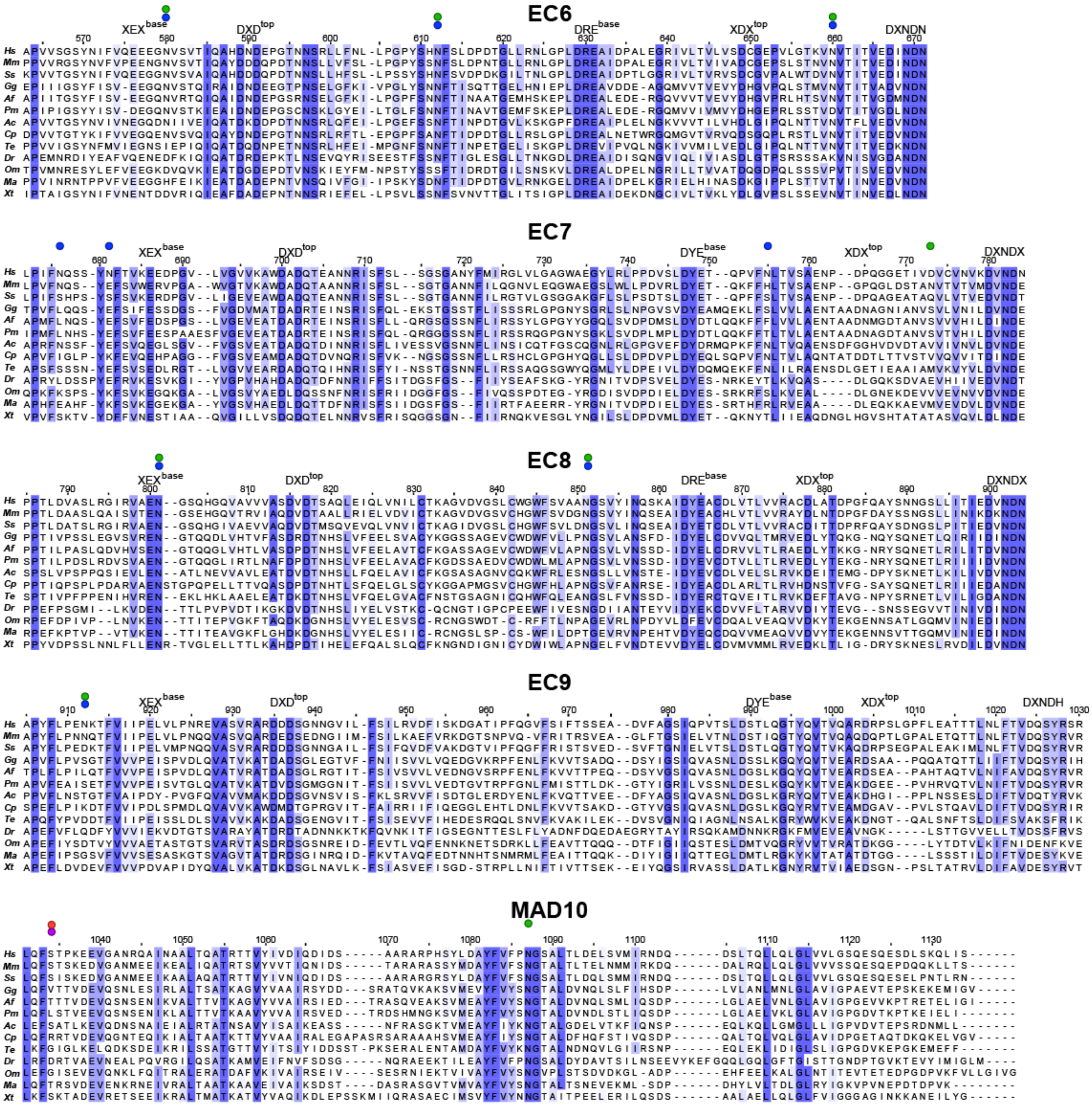

S1 Fig. Sequence alignments of individual EC repeats of PCDH24. Multiple sequence alignments comparing each EC repeat and MAD10 of PCDH24 from 13 different species. Each alignment is colored by percent identity, with white being the lowest percent identity and dark blue being the highest. Sites of N-linked (blue in human, green in mouse) and O-linked (purple in human, red in mouse) glycosylation are denoted by a colored circle. An asterisk (*) indicates sites Y67 and Y71 mutated in binding assays. Secondary structure elements observed in the crystal structures of *hs* PCDH24 EC1-2 and *mm* PCDH24 EC1-3 are illustrated below the respective repeats. Residues at the antiparallel homophilic *hs* PCDH24 EC1-2 II interface (>30% buried surface area) are denoted by a light blue bar. Calcium-binding motifs are indicated above the sequences, which are numbered according to the human protein. Species are abbreviated as follows: *Homo sapiens* (*Hs*), *Mus musculus* (*Mm*), *Sus scrofa* (*Ss*), *Gallus gallus* (*Gg*), *Aptenodytes forsteri* (*Af*), *Parus major* (*Pm*), *Anolis carolinensis* (*Ac*), *Crocodylus porosus* (*Cp*), *Thamnophis elegans* (*Te*), *Danio rerio* (*Dr*), *Oryzias melastigma* (*Om*), *Mastacembelus armatus* (*Ma*), *and Xenopus tropicalis* (*Xt*). Species were chosen based on sequence availability, coverage of entire extracellular domain, and taxonomical diversity. Accession numbers and species can be found in S2 Table.
