# Supplementary material for: Heterophilic and homophilic cadherin interactions in intestinal intermicrovillar links are species dependent
Source: PLoS Biol. 2021 Dec 6;19(12):e3001463. doi: 10.1371/journal.pbio.3001463 (PMC8691648; doi:10.1371/journal.pbio.3001463)
Supplement: S2 Fig — Multiple sequence alignments comparing each EC repeat of CDHR5 from 13 different species, shown as in S1 Fig. An asterisk (*) indicates site R84 mutated in binding assays. An arrow indicates the end of EC1-EC4 protein fragments used in binding assays. Secondary structure elements observed in the crystal structures of hs CDHR5 EC1-2 are illustrated below the respective repeats. Calcium-binding motifs are indicated above the sequences, which are numbered according to the human protein. Species are abbreviated as follows: Homo sapiens (Hs), Mus musculus (Mm), Sus scrofa (Ss), Gallus gallus (Gg), Aptenodytes forsteri (Af), Parus major (Pm), Anolis carolinensis (Ac), Crocodylus porosus (Cp), Thamnophis elegans (Te), Danio rerio (Dr), Oryzias melastigma (Om), Mastacembelus armatus (Ma), and Xenopus tropicalis (Xt). Species were chosen based on sequence availability and taxonomical diversity. Accession numbers and species can be found in S2 Table. CDHR5, cadherin-related family member 5; EC, extracellular cadherin. (PDF) [file pbio.3001463.s002.pdf]

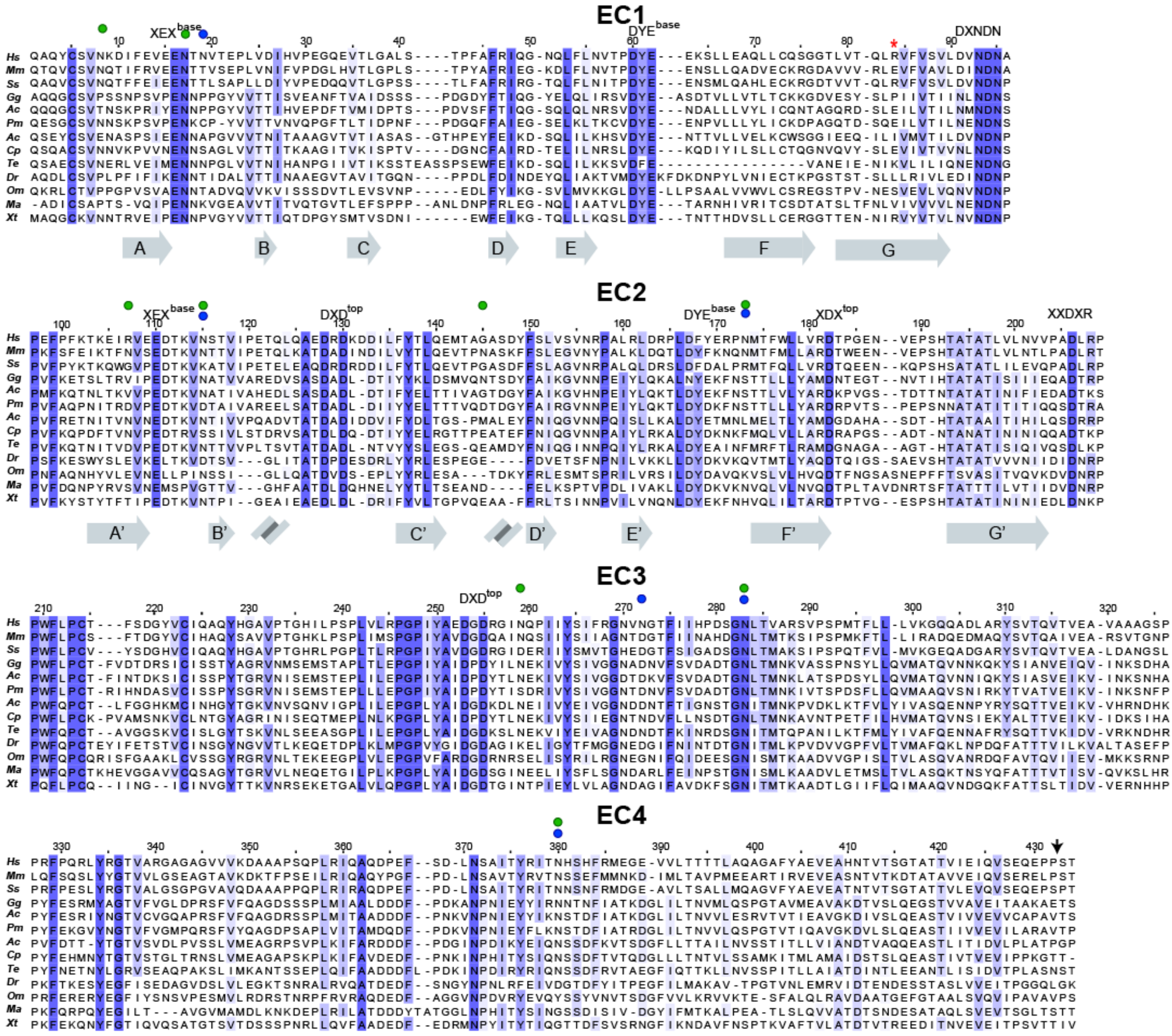

S2 Fig. Sequence alignments of individual EC repeats of CDHR5. Multiple sequence alignments comparing each EC repeat of CDHR5 from 13 different species, shown as in S1 Fig. An asterisk (*) indicates site R84 mutated in binding assays. An arrow indicates the end of EC1-EC4 protein fragments used in binding assays. Secondary structure elements observed in the crystal structures of *hs* CDHR5 EC1-2 are illustrated below the respective repeats. Calcium-binding motifs are indicated above the sequences, which are numbered according to the human protein. Species are abbreviated as follows: *Homo sapiens* (*Hs*), *Mus musculus* (*Mm*), *Sus scrofa* (*Ss*), *Gallus gallus* (*Gg*), *Aptenodytes forsteri* (*Af*), *Parus major* (*Pm*), *Anolis carolinensis* (*Ac*), *Crocodylus porosus* (*Cp*), *Thamnophis elegans* (*Te*), *Danio rerio* (*Dr*), *Oryzias melastigma* (*Om*), *Mastacembelus armatus* (*Ma*), *and Xenopus tropicalis* (*Xt*). Species were chosen based on sequence availability and taxonomical diversity. Accession numbers and species can be found in S2 Table.
