# Supplementary material for: Heterophilic and homophilic cadherin interactions in intestinal intermicrovillar links are species dependent
Source: PLoS Biol. 2021 Dec 6;19(12):e3001463. doi: 10.1371/journal.pbio.3001463 (PMC8691648; doi:10.1371/journal.pbio.3001463)
Supplement: S3 Fig — (A) Alignment of the processed N-terminal sequences of Cr-2 protocadherins hs PCDH24, mm PCDH24, hs CDH23, and hs PCDH21 and of 2 classical cadherins, hs CDH1 and hs CDH2. Calcium-binding sites are labeled and boxed in red in the sequence to show the additional calcium-binding sites in Cr-2 protocadherins not present in classical cadherins. Residue W2 involved in classical cadherin binding is boxed in pink. (B) Alignments of the processed sequences of EC1 for hs CDHR5, mm CDHR5, and hs PCDH15. Calcium-binding sites are labeled. The 2 cysteine residues that form a disulfide bond between β-strands A and F are highlighted by a red box. CDH1, Cadherin-1; CDH2, Cadherin-2; CDH23, Cadherin-23; CDHR5, cadherin-related family member 5; PCDH15, protocadherin-15; PCDH21, protocadherin-21; PCDH24, protocadherin-24. (PDF) [file pbio.3001463.s003.pdf]

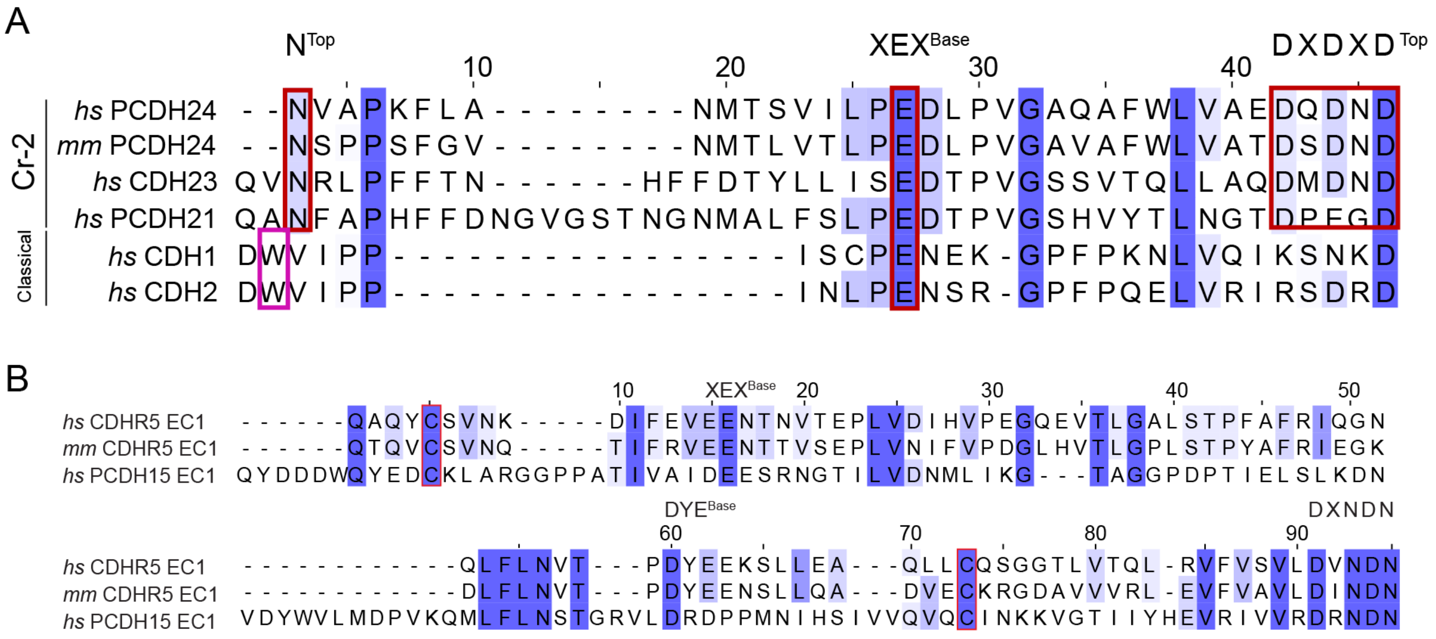

S3 Fig. Comparison of PCDH24 and CDHR5 N-termini with other cadherins. (**A**) Alignment of the processed N-terminal sequences of Cr-2 protocadherins *hs* PCDH24, *mm* PCDH24, *hs* CDH23 and *hs* PCDH21, and of two classical cadherins, *hs* CDH1 and *hs* CDH2. Calcium-binding sites are labeled and boxed in red in the sequence to show the additional calcium-bindings sites in Cr-2 protocadherins not present in classical cadherins. Residue W2 involved in classical cadherin binding is boxed in pink. (**B**) Alignments of the processed sequences of EC1 for *hs* CDHR5, *mm* CDHR5, and *hs* PCDH15. Calcium-binding sites are labeled. The two cysteine residues that form a disulfide bond between β-strands A and F are highlighted by a red box.
