# Supplementary material for: Heterophilic and homophilic cadherin interactions in intestinal intermicrovillar links are species dependent
Source: PLoS Biol. 2021 Dec 6;19(12):e3001463. doi: 10.1371/journal.pbio.3001463 (PMC8691648; doi:10.1371/journal.pbio.3001463)
Supplement: S4 Fig — (A) Extended F-G loop of EC2 in the hs PCDH24 EC1-2 I structure with the disulfide bond C187–C201. Electron density shown at 1.0 σ with a carve radius of 1.6 Å. This loop is also present in the hs PCDH24 EC1-2 II and mm PCDH24 EC1-3 structures. (B) Sugars (orange) from N-glycosylation at p.N9 in EC1 of the hs PCDH24 EC1-2 II structure. Electron density shown at 0.9 σ with a carve radius of 1.6 Å. (C) The atypical extended N-terminus seen in mm PCDH24 EC1. Electron density shown at 1.0 σ with a carve radius of 1.8 Å. (D) Disulfide bond between residue C5 in β-strand A and residue C73 in β-strand F of the hs CDHR5 EC1-2 structure. Electron density shown at 1.0 σ with a carve radius of 1.6 Å. CDHR5, cadherin-related family member 5; PCDH24, protocadherin-24. (PDF) [file pbio.3001463.s004.pdf]

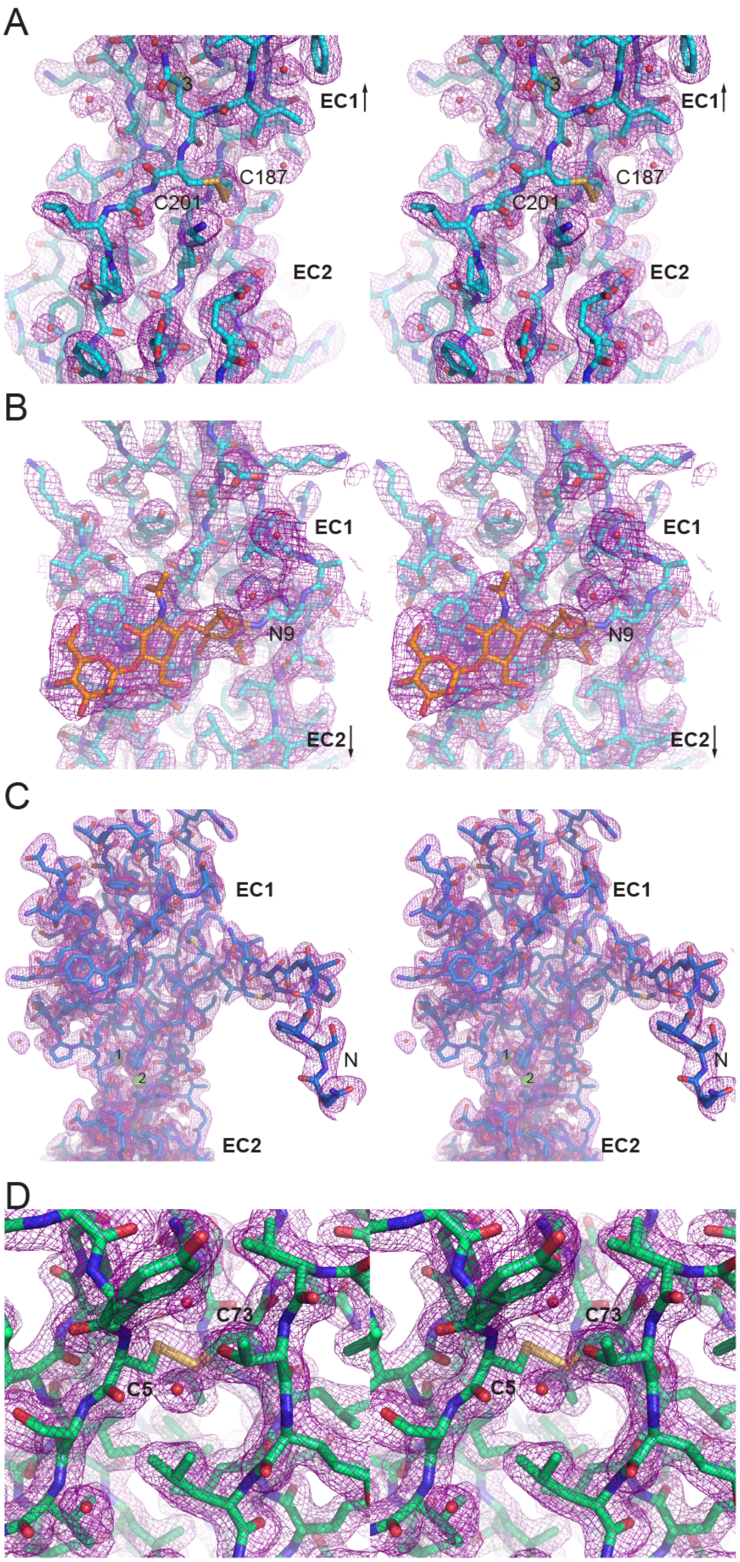

S4 Fig. Stereo views of electron density maps (2Fo –Fc) for unique elements of *hs* PCDH24 EC1-2 I, *hs* PCDH24 EC1-2 II, *mm* PCDH24 EC1-3 and *hs* CDHR5 EC1-2. (**A**) Extended F-G loop of EC2 in the *hs* PCDH24 EC1-2 I structure with the disulfide bond C187 - C201. Electron density shown at 1.0 σ with a carve radius of 1.6 Å. This loop is also present in the *hs* PCDH24 EC1-2 II and *mm* PCDH24 EC1-3 structures. (**B**) Sugars (orange) from N-glycosylation at p.N9 in EC1 of the *hs* PCDH24 EC1-2 II structure. Electron density shown at 0.9 σ with a carve radius of 1.6 Å. (**C**) The atypical extended N-terminus seen in *mm* PCDH24 EC1. Electron density shown at 1.0 σ with a carve radius of 1.8 Å. (**D**) Disulfide bond between residue C5 in β-strand A and residue C73 in β-strand F of the *hs* CDHR5 EC1-2 structure. Electron density shown at 1.0 σ with a carve radius of 1.6 Å.
