# Supplementary material for: Heterophilic and homophilic cadherin interactions in intestinal intermicrovillar links are species dependent
Source: PLoS Biol. 2021 Dec 6;19(12):e3001463. doi: 10.1371/journal.pbio.3001463 (PMC8691648; doi:10.1371/journal.pbio.3001463)
Supplement: S6 Fig — (A) Superposition of mm PCDH24 EC1-3 conformations taken every 5 ns from an approximately 99-ns-long trajectory (simulation Sim1; S7 Table). Repeat EC2 was used as a reference. Side, top, and bottom views are shown. Color indicates time step (red-white-blue). (B) Orientation projections illustrating the conformational freedom of the EC1-2 and EC2-3 linkers throughout equilibrium simulations. To quantify the conformational freedom of EC2 relative to EC1 (top) and of EC3 relative to EC2 (bottom), the longest principal axes of EC1 and EC2 were aligned to the z axis, and then the projections of the longest principal axes of EC2 (blue) and EC3 (red) in the x-y plane were plotted. The initial orientation of a control, CDH23 EC1-2 (PDB: 2WHV), is shown as a black dot [34]. The EC2-3 linker behavior is not dramatically different than the behavior observed for the EC1-2 linker, suggesting similar flexibility. (C) Trajectory snapshots during the slowest speed stretching simulation at 0.1 nm/ns for mm PCDH24 EC1-3 (Sim2d; S7 Table). Springs indicate position (center of mass) and direction of applied forces. Unfolding of the EC2-3 linker is observed first. End-to-end distances between the centers of mass of EC1 and EC3 are indicated for each snapshot. (D) Force versus end-to-end distance (S8 Data) for simulations of the mm PCDH24 EC1-3 monomer (Sim2b-Sim2d) at stretching speeds of 10 nm/ns (red), 1 nm/ns (blue), and 0.1 nm/ns (green). Dark and light colors indicate forces applied at opposite ends. Black arrowheads indicate time points illustrated in (C). (E) Detail of the mm PCDH24 EC2-3 linker at the force peak during the slowest stretching simulation at 0.1 nm/ns (Sim2d; S7 Table). Residues involved in two-step unbinding from calcium ions are shown. CDH23, Cadherin-23; PCDH24, protocadherin-24. (PDF) [file pbio.3001463.s006.pdf]

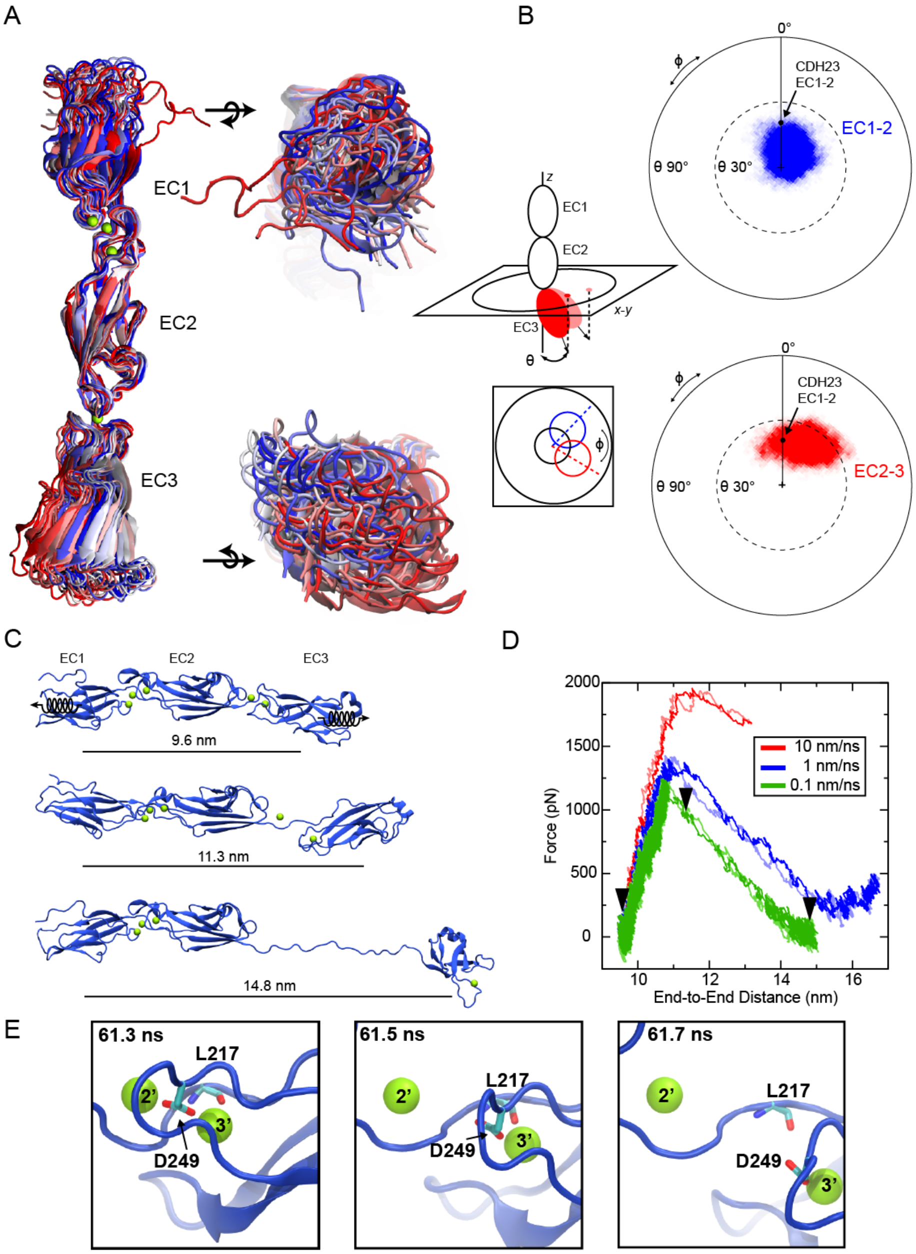

S6 Fig. Equilibrium and stretching simulations of *mm* PCDH24 EC1-3. (**A**) Superposition of *mm* PCDH24 EC1-3 conformations taken every 5 ns from a ~99-ns-long trajectory (simulation Sim1, S7 Table). Repeat EC2 was used as a reference. Side, top, and bottom views are shown. Color indicates time step (red-white-blue). (**B**) Orientation projections illustrating the conformational freedom of the EC1-2 and EC2-3 linkers throughout equilibrium simulations. To quantify the conformational freedom of EC2 relative to EC1 (top), and of EC3 relative to EC2 (bottom), the longest principal axes of EC1 and EC2 were aligned to the *z* axis and then the projections of the longest principal axes of EC2 (blue) and EC3 (red) in the *x-y* plane were plotted. The initial orientation of a control, CDH23 EC1-2 (2WHV), is shown as a black dot [34]. The EC2-3 linker behavior is not dramatically different than the behavior observed for the EC1-2 linker, suggesting similar flexibility. (**C**) Trajectory snapshots during the slowest speed stretching simulation at 0.1 nm/ns for *mm* PCDH24 EC1-3 (Sim2d, S7 Table). Springs indicate position (center of mass) and direction of applied forces. Unfolding of the EC2-3 linker is observed first. End-to-end distances between the centers of mass of EC1 and EC3 are indicated for each snapshot. (**D**) Force versus end-to-end distance (S8 Data) for simulations of the *mm* PCDH24 EC1-3 monomer (Sim2b-Sim2d) at stretching speeds of 10 nm/ns (red), 1 nm/ns (blue) and 0.1 nm/ns (green). Dark and light colors indicate forces applied at opposite ends. Black arrowheads indicate time points illustrated in (C). (**E**) Detail of the *mm* PCDH24 EC2-3 linker at the force peak during the slowest stretching simulation at 0.1 nm/ns (Sim2d, S7 Table). Residues involved in two-step unbinding from calcium ions are shown.
