# Supplementary material for: Heterophilic and homophilic cadherin interactions in intestinal intermicrovillar links are species dependent
Source: PLoS Biol. 2021 Dec 6;19(12):e3001463. doi: 10.1371/journal.pbio.3001463 (PMC8691648; doi:10.1371/journal.pbio.3001463)
Supplement: S7 Fig — (A) Detail of superposed EC1 N-termini from structures hs PCDH24 EC1-2 I (blue) and mm CDH23 EC1-2 (gray; PDB: 2WHV). Both have a calcium ion at the calcium-binding site 0, a feature of Cr-2 protocadherins. (B) Detail of superposed EC1 N-termini of mm PCDH24 (blue) and hs CDH1 (gray; PDB: 2O72). Both have N-termini protruding away from the monomer to interact with another neighboring EC1, as shown in (C) for hs CDH1 and (D) for mm PCDH24. (C, D) Surface representations of hs CDH1 and mm PCDH24, respectively. Highlighted in yellow is β-strand A with W2 from its binding partner. Residues 25, 26, and 27 are not shown to facilitate visualization of the tryptophan binding pocket. Highlighted in purple is the N-terminus of mm PCDH24 from a neighboring protomer in the crystal lattice (Fig 2D). (E) Detail of hs CDHR5 EC1 (green) and mm PCDH15 EC1 (purple) superposed. Disulfide bonds are shown in orange and yellow. PCDH15 has longer loops and a longer N-terminus, with a helix that interacts with CDH23. CDH1, Cadherin-1; CDH23, Cadherin-23; CDHR5, cadherin-related family member 5; PCDH15, protocadherin-15; PCDH24, protocadherin-24. (PDF) [file pbio.3001463.s007.pdf]

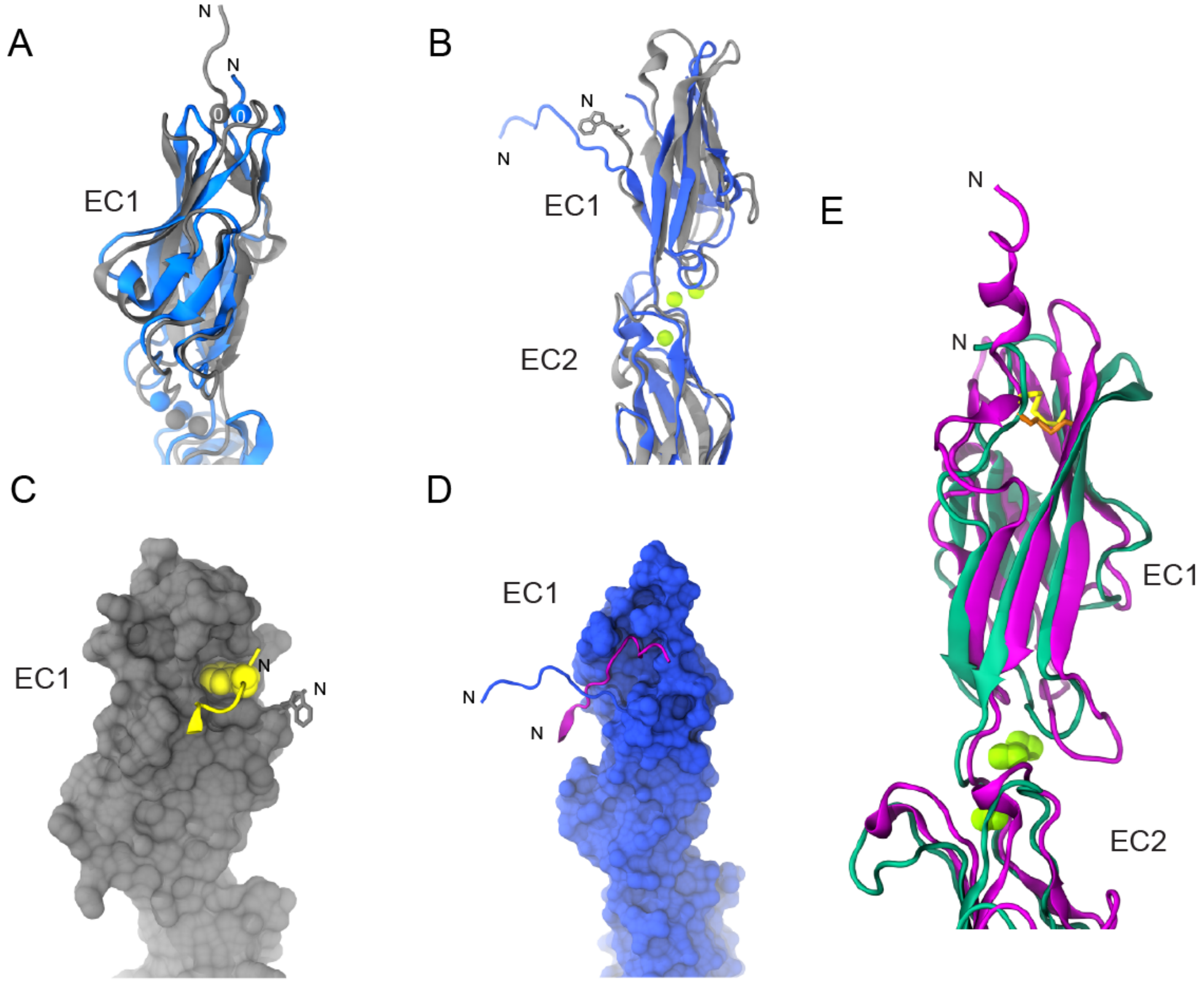

S7 Fig. Structural comparison of PCDH24 and CDHR5 EC1 repeats with other cadherins. (**A**) Detail of superposed EC1 N-termini from structures *hs* PCDH24 EC1-2 I (blue) and *mm* CDH23 EC1-2 (grey; PDB: 2WHV). Both have a calcium ion at the calcium-binding site 0, a feature of Cr-2 protocadherins. (**B**) Detail of superposed EC1 N-termini of *mm* PCDH24 (blue) and *hs* CDH1 (grey; PDB: 2O72). Both have N-termini protruding away from the monomer to interact with another neighboring EC1, as shown in (C) for *hs* CDH1 and (D) for *mm* PCDH24. (**C-D**) Surface representations of *hs* CDH1 and *mm* PCDH24, respectively. Highlighted in yellow is β-strand A with W2 from its binding partner. Residues 25, 26, and 27 are not shown to facilitate visualization of the tryptophan binding pocket. Highlighted in purple is the N-terminus of *mm* PCDH24 from a neighboring protomer in the crystal lattice (Fig 2D). (**E**) Detail of *hs* CDHR5 EC1 (green) and *mm* PCDH15 EC1 (purple) superposed. Disulfide bonds are shown in orange and yellow. PCDH15 has longer loops and a longer N-terminus, with a helix that interacts with CDH23.
