# Supplementary material for: Heterophilic and homophilic cadherin interactions in intestinal intermicrovillar links are species dependent
Source: PLoS Biol. 2021 Dec 6;19(12):e3001463. doi: 10.1371/journal.pbio.3001463 (PMC8691648; doi:10.1371/journal.pbio.3001463)
Supplement: S8 Fig — (A-D) Ribbon diagram of 2 monomers showing a crystal contact between chains A and D with an interface area of 979.2 Å2. The arrangement is antiparallel, likely describing a possible trans interface (A). Crystal contacts in the entire asymmetric unit include 4 additional interfaces with areas of 173.6 Å2 (chains A and B), 231.1 Å2 (chains A and C), 255.3 Å2 (chains D and B), and 307.2 Å2 (chains D and C). Two additional interfaces between chains A and C (387.4 Å2 and 507.3 Å2) are shown in (C) and (D). Equivalent interfaces for the one shown in (A) between chains B and C, and those shown in (C, D) between chains D and B, have similar interface areas of 938.6 Å2, 390.8 Å2, and 515.1 Å2, respectively, but are not shown. PCDH24, protocadherin-24. (PDF) [file pbio.3001463.s008.pdf]

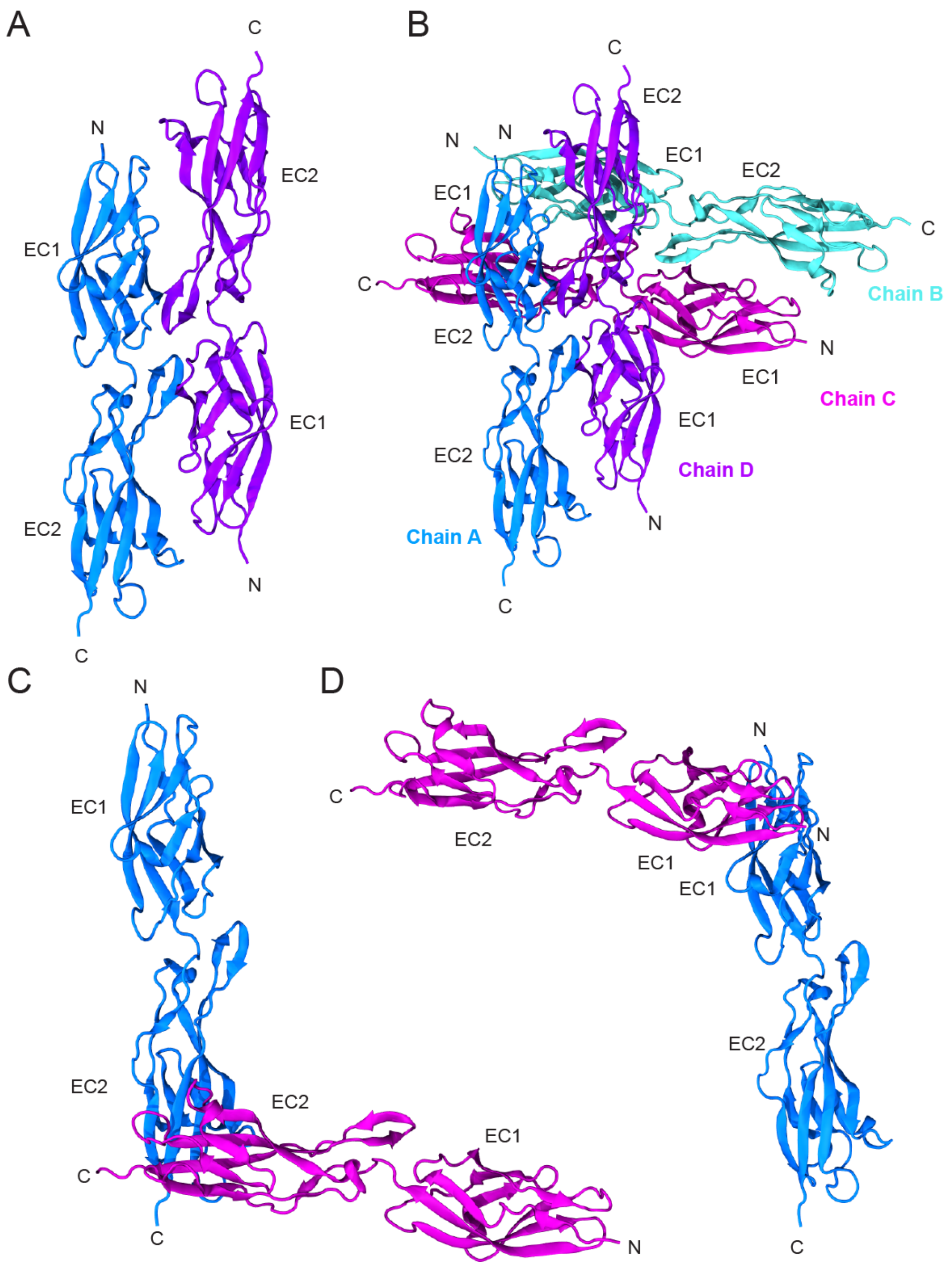

S8 Fig. Crystal contacts in the *hs* PCDH24 EC1-2 I structure. (**A-D**) Ribbon diagram of two monomers showing a crystal contact between chains A and D with an interface area of 979.2 Å^2. The arrangement is antiparallel, likely describing a possible *trans* interface (A). Crystal contacts in the entire asymmetric unit include four additional interfaces with areas of 173.6 Å^2 (chains A and B), 231.1 Å^2 (chains A and C), 255.3 Å^2 (chains D and B), and 307.2 Å^2 (chains D and C). Two Additional interfaces between chains A and C (387.4 Å^2 and 507.3 Å^2) are shown in (C) and (D). Equivalent interfaces for the one shown in (A) between chains B and C, and those shown in (C, D) between chains D and B, have similar interface areas of 938.6 Å^2, 390.8 Å^2, and 515.1 Å^2, respectively, but are not shown.
