# Supplementary material for: Heterophilic and homophilic cadherin interactions in intestinal intermicrovillar links are species dependent
Source: PLoS Biol. 2021 Dec 6;19(12):e3001463. doi: 10.1371/journal.pbio.3001463 (PMC8691648; doi:10.1371/journal.pbio.3001463)
Supplement: S9 Fig — (A) Force versus end-to-end distance for simulations of the largest crystallographic trans interface (S8A Fig) observed for the hs PCDH24 EC1-2 I structure (simulations Sim3b-Sim3d; S7 Table, S9 Data). Forced unbinding simulations were carried out at stretching speeds of 10 nm/ns (red), 1 nm/ns (blue), and 0.1 nm/ns (green, 0.4-ns running average in light green). (B) Snapshots of unbinding trajectory during stretching simulation at 0.1 nm/ns (Sim3d; S7 Table). Springs indicate position and direction of applied forces. Top panel shows complex at the beginning of the simulation; other panels show snapshots at 3 time points indicated with black arrow heads in (A). End-to-end distance indicates the distance between the stretched atoms on the C-termini of EC2. (C) Force versus end-to-end distance for simulations of the largest crystallographic trans interface (S10A Fig) observed for the hs PCDH24 EC1-2 II structure (simulations Sim4b-Sim4d; S7 Table, S10 Data). Forced unbinding simulations were carried out at stretching speeds of 10 nm/ns (red), 1 nm/ns (blue), and 0.1 nm/ns (green, 2-ns running average in light green). PCDH24, protocadherin-24. (PDF) [file pbio.3001463.s009.pdf]

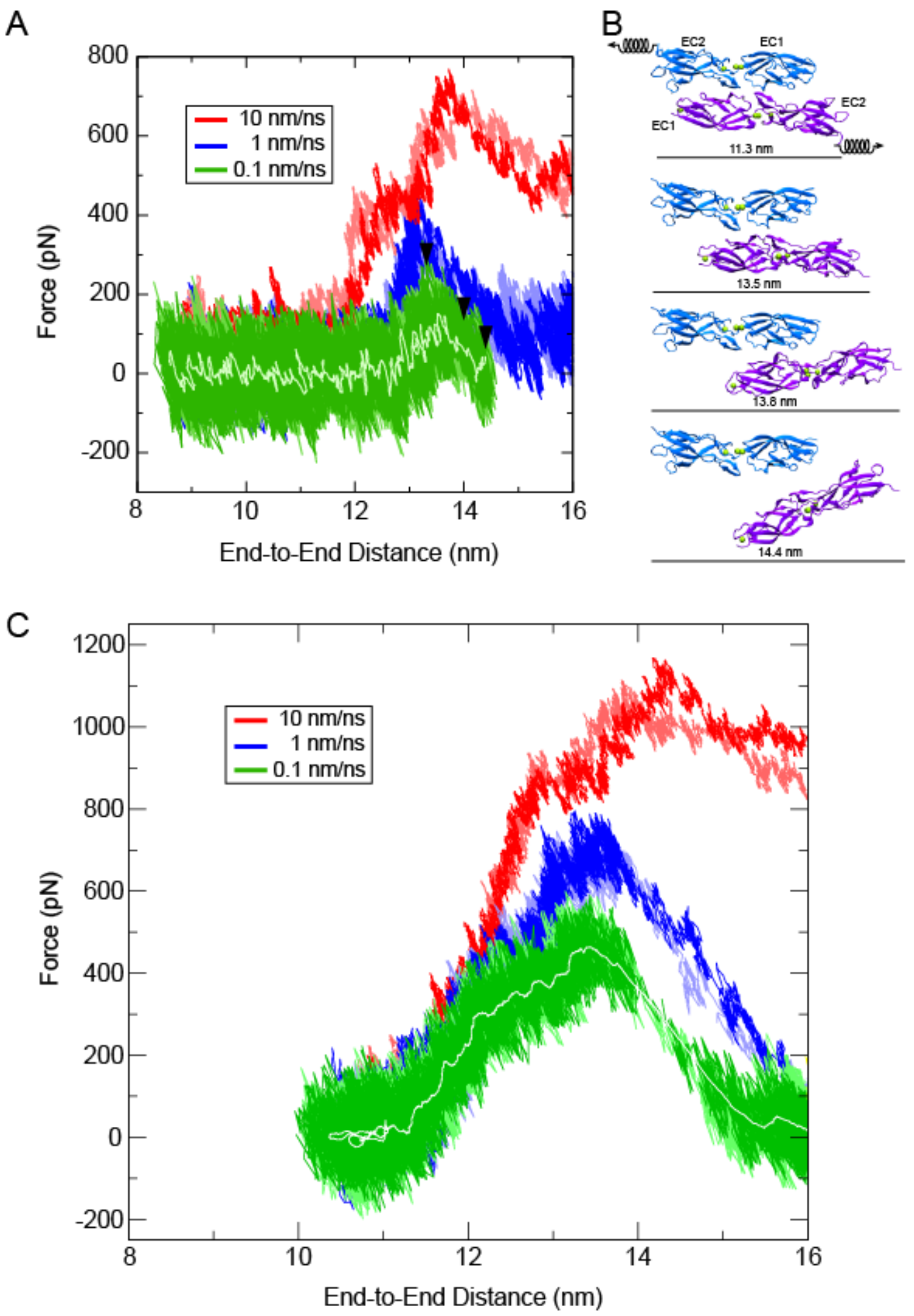

S9 Fig. Stretching simulations testing the strength of the *hs* PCDH24 EC1-2 I and II *trans* interfaces. (**A**) Force versus end-to-end distance for simulations of the largest crystallographic *trans* interface (S8A Fig) observed for the *hs* PCDH24 EC1-2 I structure (simulations Sim3b-Sim3d, S7 Table; S9 Data). Forced unbinding simulations were carried out at stretching speeds of 10 nm/ns (red), 1 nm/ns (blue), and 0.1 nm/ns (green, 0.4-ns running average in light green). (**B**) Snapshots of unbinding trajectory during stretching simulation at 0.1 nm/ns (Sim3d, S7 Table). Springs indicate position and direction of applied forces. Top panel shows complex at the beginning of the simulation, other panels show snapshots at three time points indicated with black arrow heads in (A). End-to-end distance indicates the distance between the stretched atoms on the C-termini of EC2. (**C**) Force versus end-to-end distance for simulations of the largest crystallographic *trans* interface (S10A Fig) observed for the *hs* PCDH24 EC1-2 II structure (simulations Sim4b-Sim4d, S7 Table; S10 Data). Forced unbinding simulations were carried out at stretching speeds of 10 nm/ns (red), 1 nm/ns (blue), and 0.1 nm/ns (green, 2-ns running average in light green).
