# Supplementary material for: Heterophilic and homophilic cadherin interactions in intestinal intermicrovillar links are species dependent
Source: PLoS Biol. 2021 Dec 6;19(12):e3001463. doi: 10.1371/journal.pbio.3001463 (PMC8691648; doi:10.1371/journal.pbio.3001463)
Supplement: S10 Fig — (A) Ribbon diagram of 2 monomers showing a crystal contact between chains C and D with an interface area of 1,019.3 Å2. The arrangement is antiparallel, likely describing a possible trans interface. N-glycosylation at p.N9 is shown in orange licorice. A similar arrangement is seen in chains A and B with an interface area of 1,022.8 Å2. (B) A crystal contact between chains B and D (435.5 Å2) with the F-G loops of EC2 on top of each other (black box). (C) The dimer shown in (A) forms a dimer of dimers with chains A and B to form the asymmetric unit and includes 4 additional interfaces with areas of 215.1 Å2 (chains A and D), 417.6 Å2 (chains A and C), 435.5 Å2 (chains D and B, shown in (B)), and 278.5 Å2 (chains B and C). (D) An additional trans overlap exists between chains C and D (353.1 Å2). (E, F) Two smaller interfaces between chain D and B (344.7 Å2) and chains A and D (282.1 Å2) are shown in (E) and (F), respectively. (G) Hypothetical heterotetrameric junction observed in the crystal’s asymmetric unit. PCDH24, protocadherin-24. (PDF) [file pbio.3001463.s010.pdf]

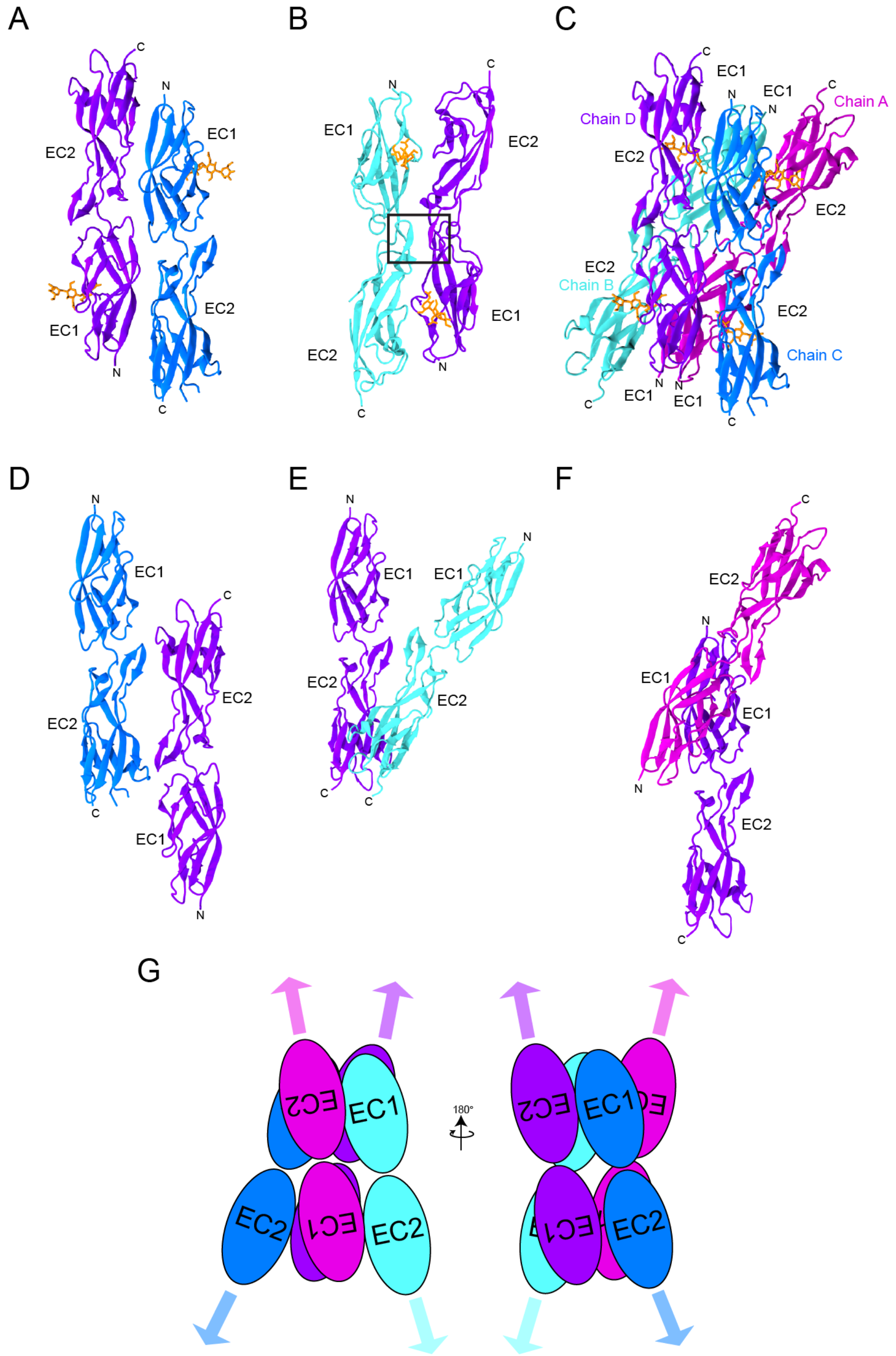

S10 Fig. Crystal contacts in the *hs* PCDH24 EC1-2 II structure. (**A**) Ribbon diagram of two monomers showing a crystal contact between chains C and D with an interface area of 1019.3 Å^2. The arrangement is antiparallel, likely describing a possible *trans* interface. N-glycosylation at p.N9 is shown in orange licorice. A similar arrangement is seen in chains A and B with an interface area of 1022.8 Å^2. (**B**) A crystal contact between chains B and D (435.5 Å^2) with the F-G loops of EC2 on top of each other (black box). (**C**) The dimer shown in (A) forms a dimer of dimers with chains A and B to form the asymmetric unit and includes four additional interfaces with areas of 215.1 Å^2 (chains A and D), 417.6 Å^2 (chains A and C), 435.5 Å^2 (chains D and B, shown in (B)), and 278.5 Å^2 (chains B and C). (**D**) An additional *trans* overlap exists between chains C and D (353.1 Å^2). (**E-F**) Two smaller interfaces between chain D and B (344.7 Å^2), and chains A and D (282.1 Å^2) are shown in (E) and (F) respectively. (**G**) Hypothetical heterotetrameric junction observed in the crystal's asymmetric unit.
