# Supplementary material for: Heterophilic and homophilic cadherin interactions in intestinal intermicrovillar links are species dependent
Source: PLoS Biol. 2021 Dec 6;19(12):e3001463. doi: 10.1371/journal.pbio.3001463 (PMC8691648; doi:10.1371/journal.pbio.3001463)
Supplement: S11 Fig — (A) Crystal contacts show a potential cis trimer formed by 3 parallel monomers. The interface area between 2 monomers is 1,352.6 Å2. Two monomers are shown as ribbons, while the third one is shown in molecular surface representation. (B) Detail of trimer seen from EC1 (top) shows that the extended N-terminal strands interlace between monomers. (C) Crystal contacts show an antiparallel trans dimer with an interface area of 1,221.2 Å2. (D) Taken together, the potential cis and trans interfaces form a large complex with 2 cis trimers forming an antiparallel trans dimer. PCDH24, protocadherin-24. (PDF) [file pbio.3001463.s011.pdf]

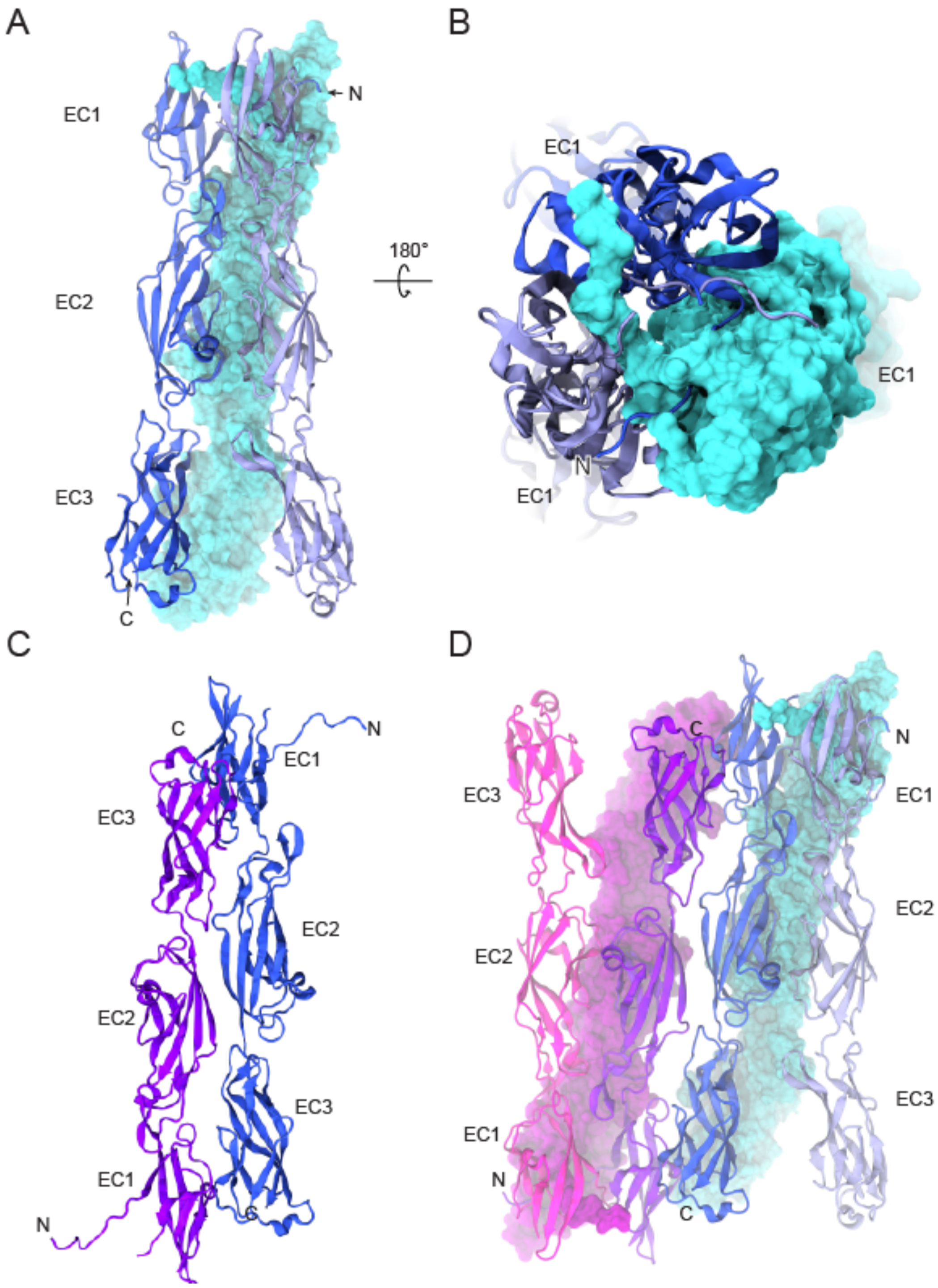

S11 Fig. Crystal contacts in the *mm* PCDH24 EC1-3 structure. (**A**) Crystal contacts show a potential *cis* trimer formed by three parallel monomers. The interface area between two monomers is 1352.6 $Å^2$. Two monomers are shown as ribbons while the third one is shown in molecular surface representation. (**B**) Detail of trimer seen from EC1 (top) shows that the extended N-terminal strands interlace between monomers. (**C**) Crystal contacts show an antiparallel *trans* dimer with an interface area of 1221.2 $Å^2$. (**D**) Taken together, the potential *cis* and *trans* interfaces form a large complex with two *cis* trimers forming an antiparallel *trans* dimer.
