# Supplementary material for: Heterophilic and homophilic cadherin interactions in intestinal intermicrovillar links are species dependent
Source: PLoS Biol. 2021 Dec 6;19(12):e3001463. doi: 10.1371/journal.pbio.3001463 (PMC8691648; doi:10.1371/journal.pbio.3001463)
Supplement: S12 Fig — (A) Surface representation of mm PCDH24 EC1-3 structure with residues colored according to sequence conservation determined using Consurf and a sequence alignment including over 94 species (S5 Table). Teal colors indicate residues that are least conserved, while magenta indicates residues that are most conserved among species. (B) Transparent surface representation of mm PCDH24 EC1-3 with most conserved residues shown as an opaque magenta surface. Protein core is conserved. PCDH24, protocadherin-24. (PDF) [file pbio.3001463.s012.pdf]

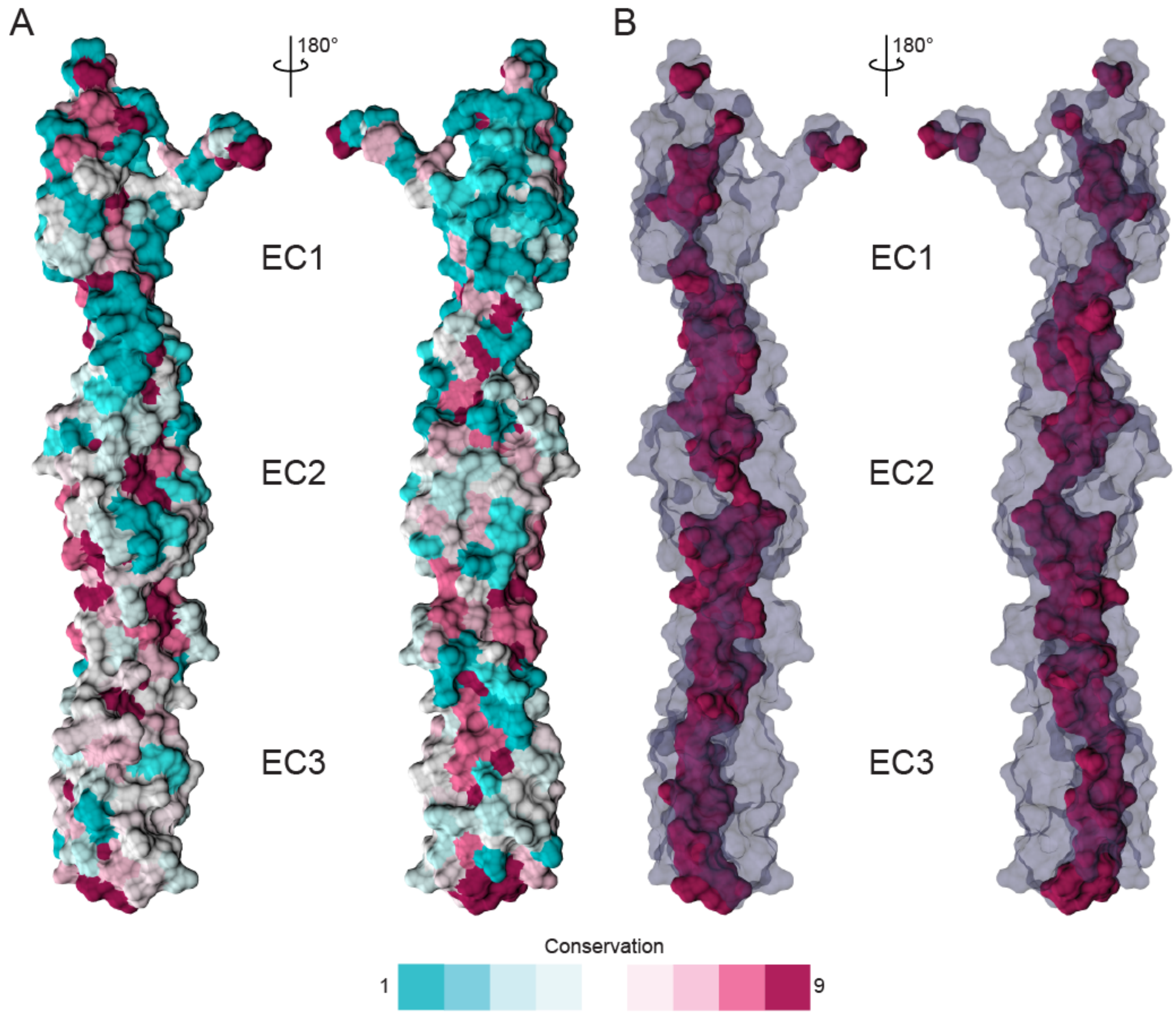

S12 Fig. Sequence conservation of PCDH24 EC1-3. (**A**) Surface representation of *mm* PCDH24 EC1-3 structure with residues colored according to sequence conservation determined using Consurf and a sequence alignment including over 94 species (S5 Table). Teal colors indicate residues that are least conserved while magenta indicates residues that are most conserved among species. (**B**) Transparent surface representation of *mm* PCDH24 EC1-3 with most conserved residues shown as an opaque magenta surface. Protein core is conserved.
