# Supplementary material for: Heterophilic and homophilic cadherin interactions in intestinal intermicrovillar links are species dependent
Source: PLoS Biol. 2021 Dec 6;19(12):e3001463. doi: 10.1371/journal.pbio.3001463 (PMC8691648; doi:10.1371/journal.pbio.3001463)
Supplement: S13 Fig — (A-H) Protein G beads coated with the Fc-tagged full-length extracellular domain of hs PCDH24 (A) and its C-terminal truncation versions (B-H). Images show bead aggregation observed at the start of the experiment (T0), after 30 min (T30), after 60 min (T60) followed by rocking for 1 min (R1), all in the presence of 2 mM CaCl2. Bar– 500 μm. (I) Protein G beads coated with the Fc-tagged full-length extracellular domain of hs PCDH24 in the presence of 2 mM EDTA, shown as in (A). PCDH24, protocadherin-24. (PDF) [file pbio.3001463.s013.pdf]

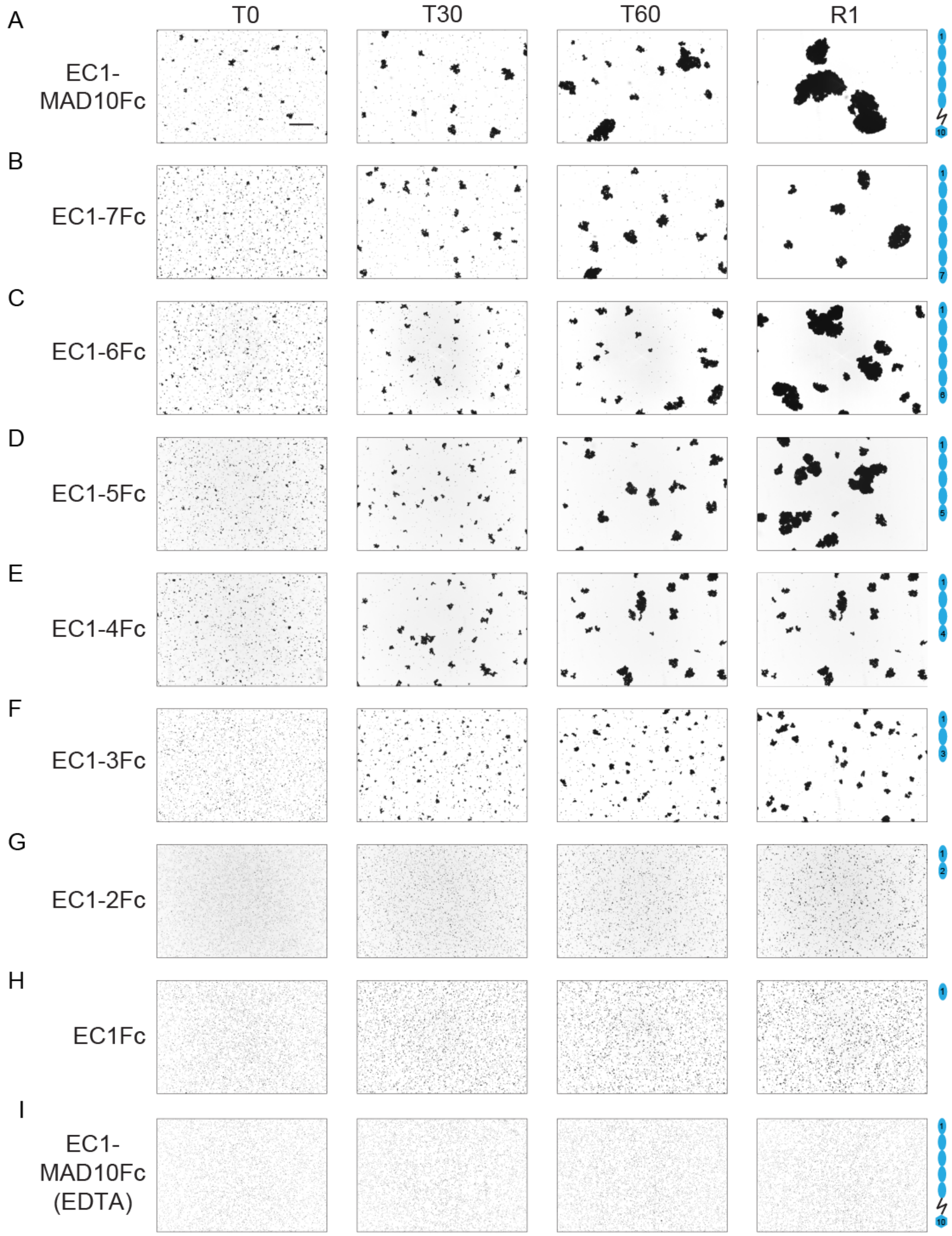

S13 Fig. Homophilic binding assays of *hs* PCDH24 at various time points. (**A-H**) Protein G beads coated with the Fc-tagged full-length extracellular domain of *hs* PCDH24 (A) and its C-terminal truncation versions (B-H). Images show bead aggregation observed at the start of the experiment (T0), after 30 min (T30), after 60 min (T60) followed by rocking for 1 min (R1), all in the presence of 2 mM $CaCl_2$. Bar – 500 µm. (**I**) Protein G beads coated with the Fc-tagged full-length extracellular domain of *hs* PCDH24 in the presence of 2 mM EDTA, shown as in (A).
