# Supplementary material for: Heterophilic and homophilic cadherin interactions in intestinal intermicrovillar links are species dependent
Source: PLoS Biol. 2021 Dec 6;19(12):e3001463. doi: 10.1371/journal.pbio.3001463 (PMC8691648; doi:10.1371/journal.pbio.3001463)
Supplement: S14 Fig — (A-D) Protein G beads coated with the Fc-tagged full-length cadherin extracellular domain of mm CDHR5 (A) and its C-terminal truncation versions (B-D). Images show the aggregation observed at the start of experiment (T0), after 60 min (T60) followed by rocking for 1 min (R1) and 2 min (R2) in the presence of 2 mM CaCl2. Bar– 500 μm. (E) Protein G beads coated with the Fc-tagged full-length cadherin extracellular domain of mm CDHR5 in the presence of 2 mM EDTA shown as in (A). CDHR5, cadherin-related family member 5. (PDF) [file pbio.3001463.s014.pdf]

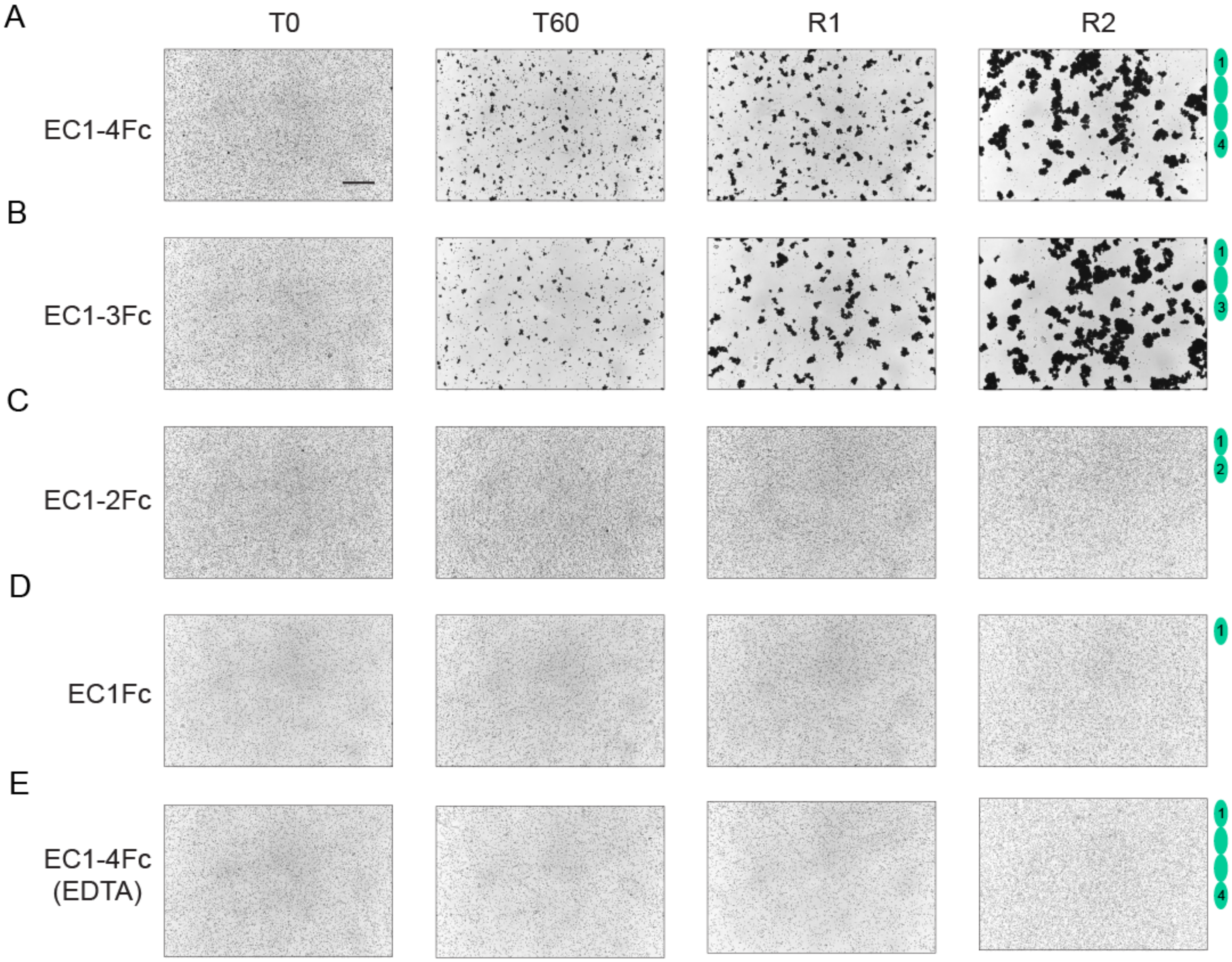

S14 Fig. Homophilic binding assays of *mm* CDHR5 at various time points. (**A-D**) Protein G beads coated with the Fc-tagged full-length cadherin extracellular domain of *mm* CDHR5 (A) and its C-terminal truncation versions (B-D). Images show the aggregation observed at the start of experiment (T0), after 60 min (T60) followed by rocking for 1 min (R1) and 2 min (R2) in the presence of 2 mM $CaCl_2$. Bar – 500 µm. (**E**) Protein G beads coated with the Fc-tagged full-length cadherin extracellular domain of *mm* CDHR5 in the presence of 2 mM EDTA shown as in (A).
