# Supplementary material for: Heterophilic and homophilic cadherin interactions in intestinal intermicrovillar links are species dependent
Source: PLoS Biol. 2021 Dec 6;19(12):e3001463. doi: 10.1371/journal.pbio.3001463 (PMC8691648; doi:10.1371/journal.pbio.3001463)
Supplement: S16 Fig — (A-F) Ribbon diagrams showing various crystal contacts between monomers of hs CDHR5 EC1-2. Interface areas are 1,433.9 Å2 (A), 530.2 Å2 (B), 364.7 Å2 (C), 347.9 Å2 (D), 132.7 Å2 (E), and 114.0 Å2 (F), respectively. Interfaces in (A) and (B) correspond to possible trans overlaps of EC1-2 and EC1-3, respectively. While hs CDHR5 does not mediate trans homophilic adhesion based on the homophilic binding assay data presented in Fig 4, these interfaces may serve as templates for the mouse CDHR5 protein. Crystal contacts in (D-F) are unlikely to be of physiological relevance due to the arrangement of the monomers. CDHR5, cadherin-related family member 5. (PDF) [file pbio.3001463.s016.pdf]

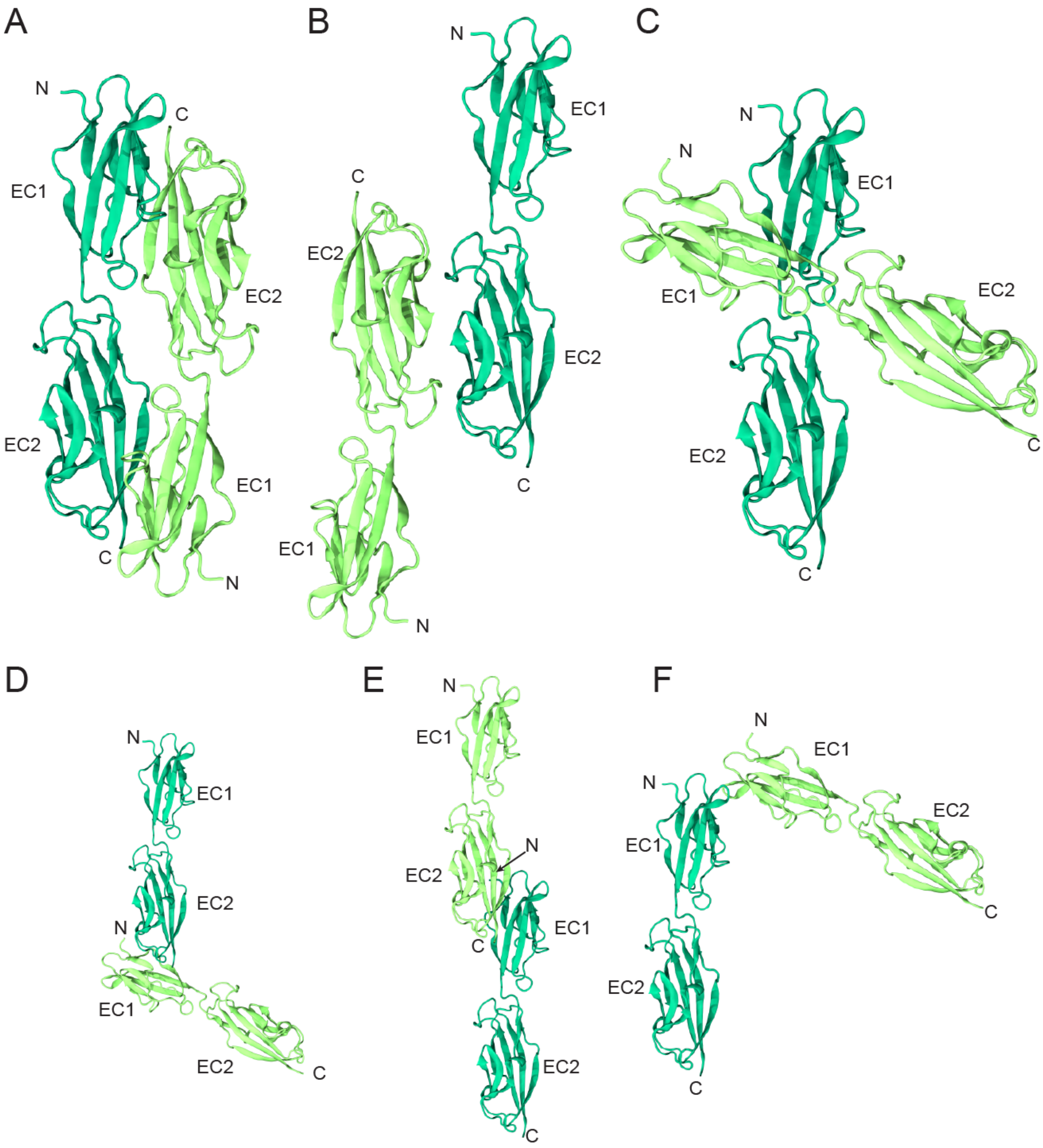

S16 Fig. Crystal contacts in the *hs* CDHR5 EC1-2 structure. (**A-F**) Ribbon diagrams showing various crystal contacts between monomers of *hs* CDHR5 EC1-2. Interface areas are 1433.9 Å^2 (A), 530.2 Å^2 (B), 364.7 Å^2 (C), 347.9 Å^2 (D), 132.7 Å^2 (E), and 114.0 Å^2 (F), respectively. Interfaces in (A) and (B) correspond to possible *trans* overlaps of EC1-2 and EC1-3, respectively. While *hs* CDHR5 does not mediate *trans* homophilic adhesion based on the homophilic binding assay data presented in Fig 4, these interfaces may serve as templates for the mouse CDHR5 protein. Crystal contacts in (D-F) are unlikely to be of physiological relevance due to the arrangement of the monomers.
