# Supplementary material for: Heterophilic and homophilic cadherin interactions in intestinal intermicrovillar links are species dependent
Source: PLoS Biol. 2021 Dec 6;19(12):e3001463. doi: 10.1371/journal.pbio.3001463 (PMC8691648; doi:10.1371/journal.pbio.3001463)
Supplement: S17 Fig — (A) Surface representation of hs CDHR5 EC1-2 structure with residues colored according to sequence conservation determined using Consurf and a sequence alignment including over 69 species (S6 Table). Teal colors indicate residues that are least conserved, while magenta indicates residues that are most conserved among species. (B) Transparent surface representation of hs CDHR5 EC1-2 with most conserved residues shown as an opaque magenta surface. CDHR5, cadherin-related family member 5. (PDF) [file pbio.3001463.s017.pdf]

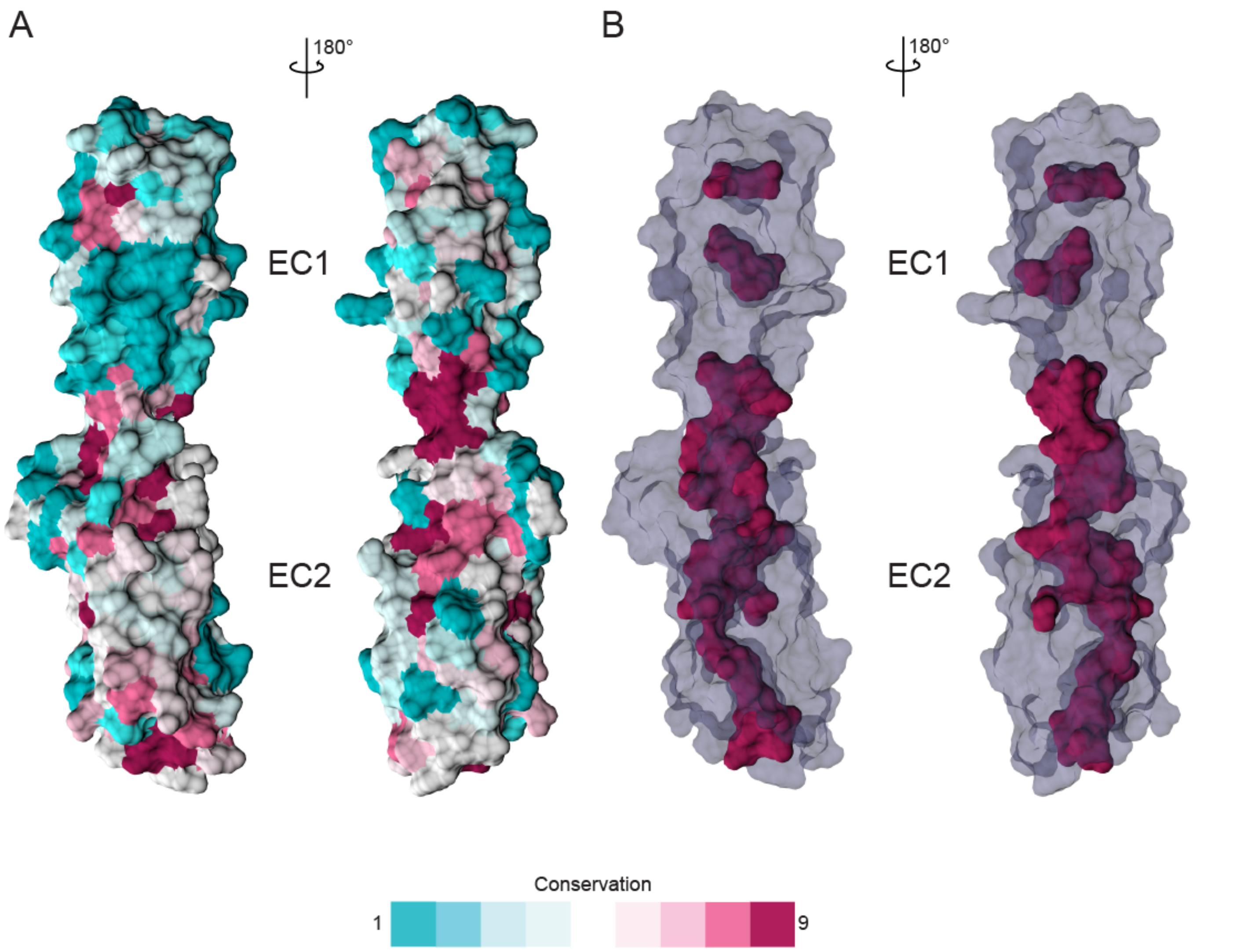

S17 Fig. Sequence conservation of CDHR5 EC1-2. (**A**) Surface representation of *hs* CDHR5 EC1-2 structure with residues colored according to sequence conservation determined using Consurf and a sequence alignment including over 69 species (S6 Table). Teal colors indicate residues that are least conserved while magenta indicates residues that are most conserved among species. (**B**) Transparent surface representation of *hs* CDHR5 EC1-2 with most conserved residues shown as an opaque magenta surface.
