# Supplementary material for: Heterophilic and homophilic cadherin interactions in intestinal intermicrovillar links are species dependent
Source: PLoS Biol. 2021 Dec 6;19(12):e3001463. doi: 10.1371/journal.pbio.3001463 (PMC8691648; doi:10.1371/journal.pbio.3001463)
Supplement: S18 Fig — (A, B) Images from binding assays of Fc-tagged hs PCDH24 (full-length extracellular domain and EC1-4Fc) mixed with the Fc-tagged full-length hs CDHR5 cadherin extracellular domain (A) and Fc-tagged mm PCDH24 (full-length extracellular domain and EC1-4Fc) mixed with Fc-tagged full-length mm CDHR5 cadherin extracellular domain (B). Green fluorescent Protein A beads are coated with Fc-tagged PCDH24 fragments and red fluorescent Protein A beads are coated with Fc-tagged CDHR5 in all panels. The white boxes indicate the regions shown in Fig 7. Images show bead aggregation observed after 60 min followed by rocking for 2 min in the presence of 2 mM CaCl2. (C-F) Images from binding assays of truncations of Fc-tagged hs CDHR5 mixed with hs PCDH24 EC1-4Fc (C), truncations of Fc-tagged hs PCDH24 mixed with hs CDHR5 EC1-4Fc (D), truncations of Fc-tagged mm CDHR5 mixed with mm PCDH24 EC1-4Fc (E), and truncations of Fc-tagged mm PCDH24 mixed with mm CDHR5 EC1-4Fc (F). Images show bead aggregation observed after 60 min followed by rocking for 2 min in the presence of 2 mM CaCl2. (G) Images from binding assays of minimum EC repeats required for heterophilic adhesion of hs and mm PCDH24 and CDHR5. The minimum units for heterophilic adhesion for the human proteins are CDHR5 EC1Fc and PCDH24 EC1-2Fc. The minimum units for heterophilic adhesion for the mouse proteins are CDHR5 EC1-2Fc and PCDH24 EC1-2Fc. Images show bead aggregation observed after 60 min followed by rocking for 2 min in the presence of 2 mM CaCl2. (H) Protein A beads coated with Fc-tagged full-length hs and mm PCDH24 and CDHR5 cadherin extracellular domains (including MAD10 for PCDH24 when indicated and without the MLD for CDHR5) in the presence of 2 mM EDTA shown as in (A, B). Bar– 500 μm. (I) Aggregate size (S11 Data) for minimum units of heterophilic adhesion of Fc-tagged hs and mm PCDH24 and CDHR5 at the start of the experiment (T0), after 60 min (T60) followed by rocking for 1 min (R1) and 2 min (R2). (J) Aggregat [file pbio.3001463.s018.pdf]

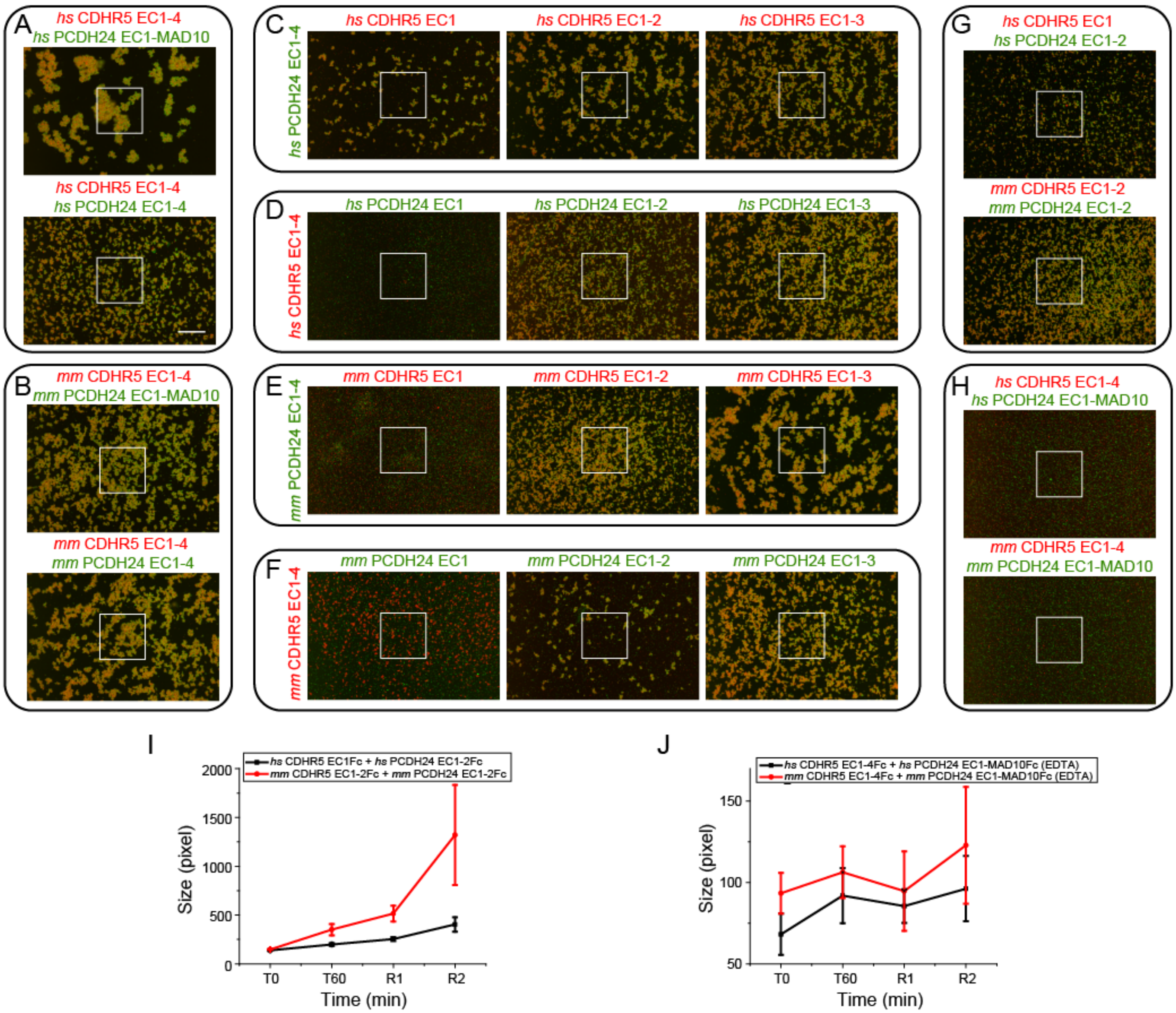


**S18 Fig. Heterophilic binding assays of *hs* and *mm* PCDH24 and CDHR5.** (**A-B**) Images from binding assays of Fc-tagged *hs* PCDH24 (full-length extracellular domain and EC1-4Fc) mixed with the Fc-tagged full-length *hs* CDHR5 cadherin extracellular domain (A), and Fc-tagged *mm* PCDH24 (full-length extracellular domain and EC1-4Fc) mixed with Fc-tagged full-length *mm* CDHR5 cadherin extracellular domain (B). Green fluorescent protein A beads are coated with Fc-tagged PCDH24 fragments and red fluorescent protein A beads are coated with Fc-tagged CDHR5 in all panels. The white boxes indicate the regions shown in Fig 6. Images show bead aggregation observed after 60 min followed by rocking for 2 min in the presence of 2 mM $CaCl_2$. (**C-F**) Images from binding assays of truncations of Fc-tagged *hs* CDHR5 mixed with *hs* PCDH24 EC1-4Fc (C), truncations of Fc-tagged *hs* PCDH24 mixed with *hs* CDHR5 EC1-4Fc (D), truncations of Fc-tagged *mm* CDHR5 mixed with *mm* PCDH24 EC1-4Fc (E), and truncations of Fc-tagged *mm* PCDH24 mixed with *mm* CDHR5 EC1-4Fc (F). Images show bead aggregation observed after 60 min followed by rocking for 2 min in the presence of 2 mM $CaCl_2$. (**G**) Images from binding assays of minimum EC repeats required for heterophilic adhesion of *hs* and *mm* PCDH24 and CDHR5. The minimum units for heterophilic adhesion for the human proteins are CDHR5 EC1Fc and PCDH24 EC1-2Fc. The minimum units for heterophilic adhesion for the mouse proteins are CDHR5 EC1-2Fc and PCDH24 EC1-2Fc. Images show bead aggregation observed after 60 minutes followed by rocking for 2 minutes in the presence of 2 mM $CaCl_2$. (**H**) Protein A beads coated with Fc-tagged full-length *hs* and *mm* PCDH24 and CDHR5 cadherin extracellular domains (including MAD10 for PCDH24 when indicated and without the mucin-like domain for CDHR5) in the presence of 2 mM EDTA shown as in (A-B). Bar – 500 µm. (**I**) Aggregate size (S11 Data) for minimum units of heterophilic adhesion of Fc-tagged *hs* and *mm* PCDH24 and CDHR5 at the start of the experiment (T0), after 60 min (T60) followed by rocking for 1 min (R1) and 2 min (R2). (**J**) Aggregate size (S12 Data) for full-length *hs* and *mm* PCDH24 and CDHR5 at the start of the experiment (T0), after 60 min (T60) followed by rocking for 1 min (R1) and 2 min (R2). Error bars in I and J are standard error of the mean (*n* indicated in S4 Table).
