# Supplementary material for: Heterophilic and homophilic cadherin interactions in intestinal intermicrovillar links are species dependent
Source: PLoS Biol. 2021 Dec 6;19(12):e3001463. doi: 10.1371/journal.pbio.3001463 (PMC8691648; doi:10.1371/journal.pbio.3001463)
Supplement: S19 Fig — Raw data from the SPR experiment performed on mm PCDH24 EC1-2Fc and mm CDHR5 EC1-2 shown in green gradient from lowest concentration (light green) to highest concentration (dark green) of mm CDHR5 EC1-2, with fits in black. The average affinity KD and rates (kon and koff) are indicated (n = 1). Values were obtained from a kinetic analysis of the raw data (S13 Data). CDHR5, cadherin-related family member 5; PCDH24, protocadherin-24; SPR, surface plasmon resonance. (PDF) [file pbio.3001463.s019.pdf]

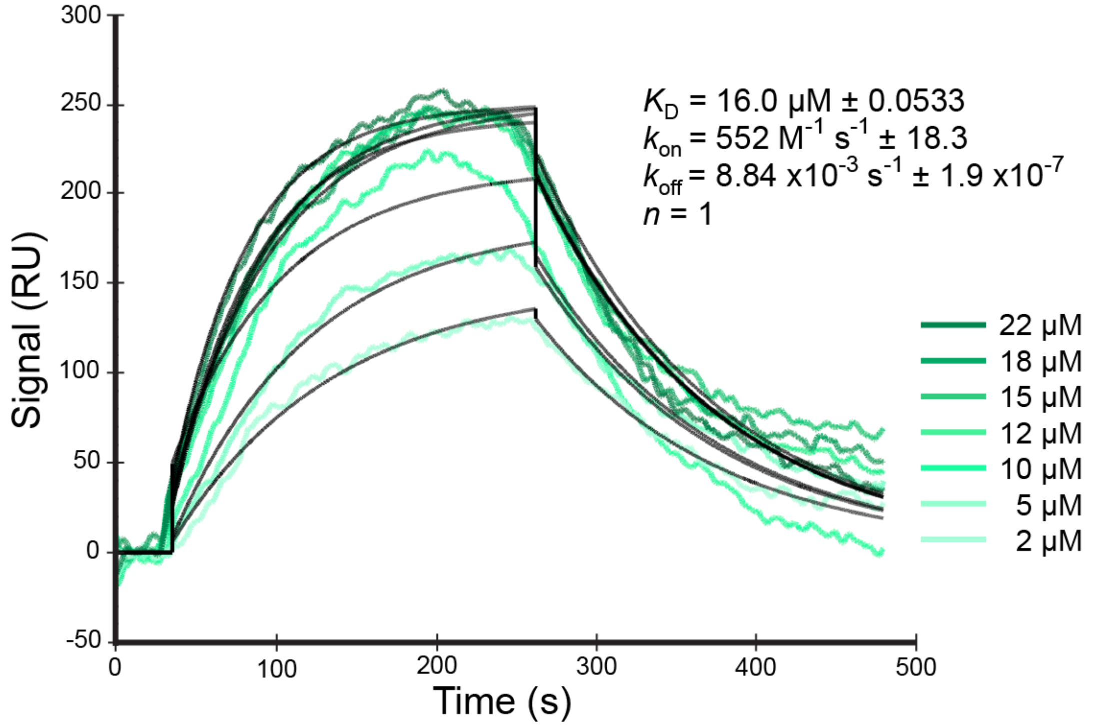

S19 Fig. Surface plasmon resonance (SPR) experiments testing the minimal adhesive unit for mouse PCDH24 and CDHR5. Raw data from the SPR experiment performed on *mm* PCDH24 EC1-2Fc and *mm* CDHR5 EC1-2 shown in green gradient from lowest concentration (light green) to highest concentration (dark green) of *mm* CDHR5 EC1-2, with fits in black. The average affinity K_D and rates (k_{on} and k_{off}) are indicated (n = 1). Values were obtained from a kinetic analysis of the raw data (S13 Data).
