# Supplementary material for: Heterophilic and homophilic cadherin interactions in intestinal intermicrovillar links are species dependent
Source: PLoS Biol. 2021 Dec 6;19(12):e3001463. doi: 10.1371/journal.pbio.3001463 (PMC8691648; doi:10.1371/journal.pbio.3001463)
Supplement: S20 Fig — Complexes were built by aligning protein fragments to monomers forming the largest antiparallel trans crystallographic interface in the hs PCDH24 EC1-2 II structure. (A) Molecular surface representation of hs PCDH24 EC1-2 (light blue) and hs CDHR5 EC1-2 (dark green) with a potential interface surface (silver) exposed. Interfacing residues are listed. Orange labels highlight residues that remained buried at the interface during a 10-ns long equilibrium simulation. Residues in hs CDHR5 EC2 are in gray as the minimum unit for heterophilic adhesion includes hs CDHR5 EC1. (B) Molecular surface representation of mm PCDH24 EC1-2 (dark blue) and mm CDHR5 EC1-2 (light green) with a potential interface surface (silver) exposed. Interfacing residues are listed. CDHR5, cadherin-related family member 5; PCDH24, protocadherin-24. (PDF) [file pbio.3001463.s020.pdf]

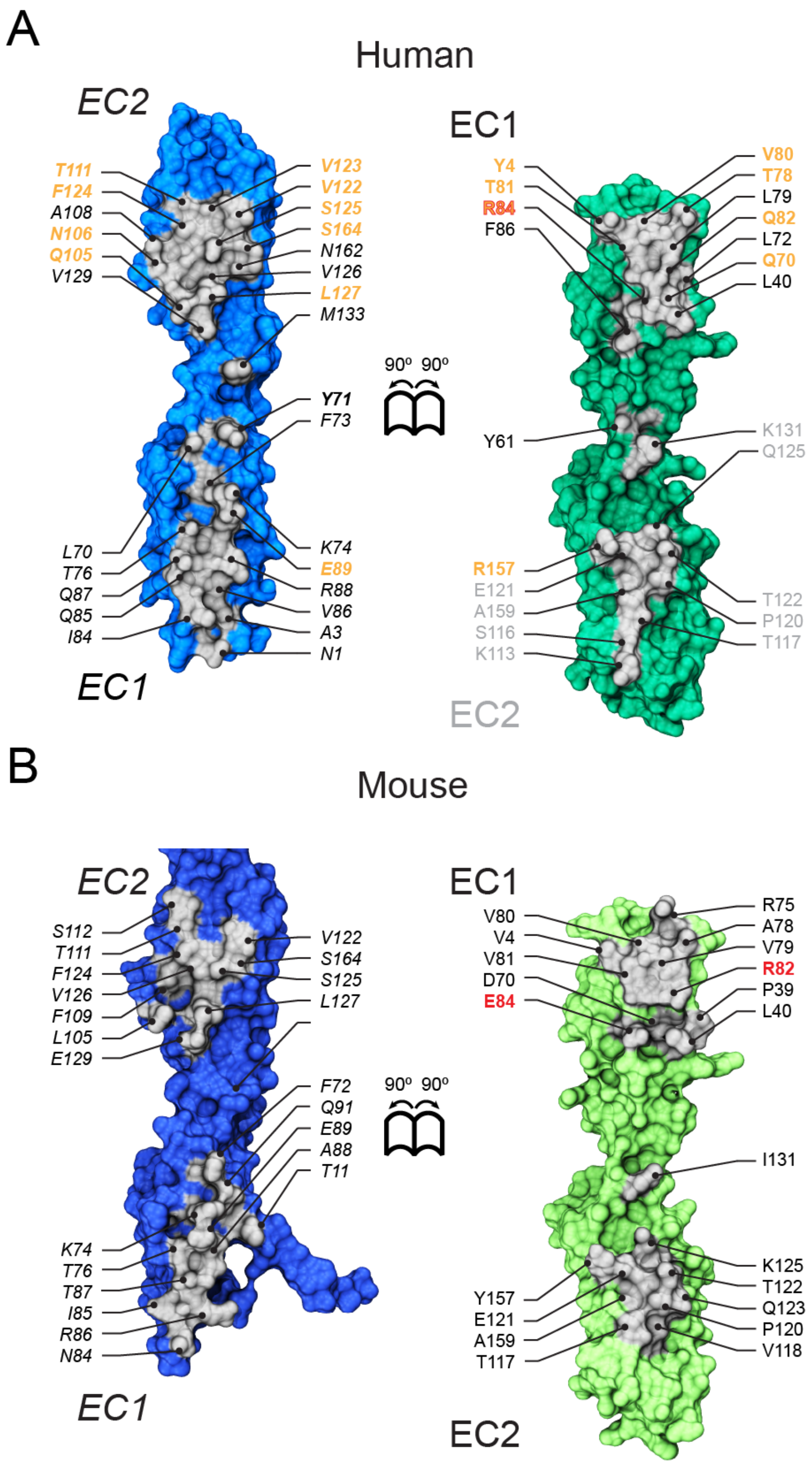

S20 Fig. Potential PCDH24 EC1-2 / CDHR5 EC1-2 complex interfaces. Complexes were built by aligning protein fragments to monomers forming the largest antiparallel *trans* crystallographic interface in the *hs* PCDH24 EC1-2 II structure. (**A**) Molecular surface representation of *hs* PCDH24 EC1-2 (light blue) and *hs* CDHR5 EC1-2 (dark green) with a potential interface surface (silver) exposed. Interfacing residues are listed. Orange labels highlight residues that remained buried at the interface during a 10-ns long equilibrium simulation. Residues in *hs* CDHR5 EC2 are in gray as the minimum unit for heterophilic adhesion includes *hs* CDHR5 EC1. (**B**) Molecular surface representation of *mm* PCDH24 EC1-2 (dark blue) and *mm* CDHR5 EC1-2 (light green) with a potential interface surface (silver) exposed. Interfacing residues are listed.
