# Supplementary material for: Heterophilic and homophilic cadherin interactions in intestinal intermicrovillar links are species dependent
Source: PLoS Biol. 2021 Dec 6;19(12):e3001463. doi: 10.1371/journal.pbio.3001463 (PMC8691648; doi:10.1371/journal.pbio.3001463)
Supplement: S1 Table — The accession numbers of the species used are listed in S2 Table. (PDF) [file pbio.3001463.s021.pdf]

S1 Table. Percent identity of EC repeats for CDH23, PCDH15, PCDH24 and CDHR5 proteins across species. The accession numbers of the species used are listed in S2 Table.

CDH23	Repeat % identity	PCDH15	Repeat % identity	PCDH24	Repeat % identity	CDHR5	Repeat % identity
1	51.4	1	57.4	1	15.8	1	9.5
2	66.3	2	56.8	2	16.8	2	12.2
3	64.3	3	56.2	3	17.7	3	14.8
4	60.7	4	47.4	4	22.3	4	4.5
5	49.5	5	46.2	5	22.6	Avg	10.3
6	44.5	6	61.8	6	20.9		
7	70.1	7	40.2	7	15.6		
8	48.2	8	31.2	8	14.8		
9	43.8	9	48.6	9	10.1		
10	59.8	10	44	MAD10	9.4		
11	50.9	11	49.5	Avg	16.6		
12	61	MAD12	54.3				
13	49.5	Avg	49.5				
14	38.5						
15	55.1						
16	40.9						
17	52.3						
18	44.4						
19	34.5						
20	36.2						
21	41.2						
22	47.7						
23	52.3						
24	49						
25	52.7						
26	43						
27	47						
MAD28	64.5						
Avg	50.7						
