# Supplementary material for: Heterophilic and homophilic cadherin interactions in intestinal intermicrovillar links are species dependent
Source: PLoS Biol. 2021 Dec 6;19(12):e3001463. doi: 10.1371/journal.pbio.3001463 (PMC8691648; doi:10.1371/journal.pbio.3001463)
Supplement: S2 Table — (PDF) [file pbio.3001463.s022.pdf]

S2 Table. Accession numbers of CDHR5, PCDH24, PCDH15, and CDH23 sequences used for alignment of EC repeats across different species.

Species Name	Abbr.	Common Name	CDHR5	PCDH24	PCDH15	CDH23
Homo sapiens	*Hs*	Human	NP_068743.3	NP_001165447.1	NP_001136235.1	NP_071407.4
Mus musculus	*Mm*	House mouse	NP_001107794.1	NP_001028536.2	NP_075604.2	NP_075859.2
Sus scrofa	*Ss*	Pig	XP_013845324.2	XP_013850328.2	XP_020929194.1*	XP_001925718.2
Gallus gallus	*Gg*	Chicken	NP_001376455.1	XP_015149366.2	NP_001038119.1	XP_015143732.2
Aptenodytes forsteri	*Af*	Emperor penguin	XP_009281978.1	XP_019329391.1	XP_009273352.1	XP_019330462.1
Parus major	*Pm*	Great tit	XP_015485075.1	XP_033373653.1	XP_033371566.1	XP_015489015.1
Anolis carolinensis	*Ac*	Green anole	XP_008106729.1	XP_008119531.1	XP_016851436.1	XP_016847668.1
Crocodylus porosus	*Cp*	Saltwater crocodile	XP_019402850.1	XP_019390868.1	XP_019394135.1	XP_019394087.1
Thamnophis elegans	*Te*	Western terrestrial garter snake	XP_032068590.1*	XP_032094958.1	XP_032087433.1	XP_032088116.1
Danio rerio	*Dr*	Zebrafish	XP_021326278.1	XP_017214654.2	NP_001012500.1	NP_999974.1
Oryzias melastigma	*Om*	Indian medaka	XP_024120964.2	XP_036069707.1	XP_036071465.1	XP_024153843.1
Mastacembelus armatus	*Ma*	Zig-zag eel	XP_026168289.1	XP_026186036.1	XP_026165600.1	XP_026165849.1
Xenopus tropicalis	*Xt*	African clawed frog	XP_031756478.1	XP_031754706.1	XP_031761776.1	XP_031762583.1

* indicates a low-quality sequence
