# Supplementary material for: Heterophilic and homophilic cadherin interactions in intestinal intermicrovillar links are species dependent
Source: PLoS Biol. 2021 Dec 6;19(12):e3001463. doi: 10.1371/journal.pbio.3001463 (PMC8691648; doi:10.1371/journal.pbio.3001463)
Supplement: S3 Table — (PDF) [file pbio.3001463.s023.pdf]

S3 Table. Accession numbers of PCDH24, CDHR5, CDH23, PCDH15, CDH1, and CDH2 sequences used for generating Fig 1B.

	Accession Number for EC1-3					
Species	PCDH24	CDHR5	CDH23	PCDH15	CDH1	CDH2
Homo sapiens	NP_001165447.1	NP_068743.3	NP_071407.4	NP_001136235.1	NP_004351.1	NP_001783.2
Mus musculus	NP_001028536.2	NP_001107794.1	NP_075859.2	NP_075604.2	NP_033994.1	NP_031690.3
Danio rerio	XP_017214654.2	XP_021326278.1	NP_999974.1	NP_001012500.1	NP_571895.1	NP_571156.2
Anolis carolensis	XP_008119531.1	XP_008106723.1	XP_016847668.1	XP_016851436.1	XP_008121673.2*	XP_008106822.1
Gallus gallus	XP_015149366.1	NP_001376455.1	XP_421595.4	NP_001038119.1	NP_001034347.2	NP_001001615.1

* indicates a low-quality sequence
