# Supplementary material for: Heterophilic and homophilic cadherin interactions in intestinal intermicrovillar links are species dependent
Source: PLoS Biol. 2021 Dec 6;19(12):e3001463. doi: 10.1371/journal.pbio.3001463 (PMC8691648; doi:10.1371/journal.pbio.3001463)
Supplement: S4 Table — (PDF) [file pbio.3001463.s024.pdf]

S4 Table. Number of biological replicates for bead aggregation assays.

Species	Protein		Time points	*n*
Human	PCDH24	EC1	T = 0, 30, 60; R = 1	4
		EC1-2	T = 0, 30, 60; R = 1	4
		EC1-3	T = 0, 30, 60; R = 1	3
		EC1-4	T = 0, 30, 60; R = 1	3
		EC1-5	T = 0, 30, 60; R = 1	3
		EC1-6	T = 0, 30, 60; R = 1	3
		EC1-7	T = 0, 30, 60; R = 1	3
		EC1-MAD10	T = 0, 30, 60; R = 1	4
		EC1-MAD10*	T = 0, 60; R = 1	3
		EC1-3	T = 0, 30, 60	4
		EC1-3 Y67A	T = 0, 30, 60	3
		EC1-3 Y71A	T = 0, 30, 60	3
		EC1-2	T = 0, 30, 60	3
		EC1-2 Y67A	T = 0, 30, 60	3
		EC1-2 Y71A	T = 0, 30, 60	3
Mouse	PCDH24	EC1-MAD10	T = 0, 60; R = 1	3
Human	CDHR5	EC1-4	T = 0, 60; R = 1, 2	3
Mouse	CDHR5	EC1	T = 0, 60; R = 1, 2	3
		EC1-2	T = 0, 60; R = 1, 2	3
		EC1-3	T = 0, 60; R = 1, 2	3
		EC1-4	T = 0, 60; R = 1, 2	3
		EC1-4 E84G	T = 0, 60; R = 1, 2	3
		EC1-4 R82G	T = 0, 60; R = 1, 2	3
Human	CDHR5 EC1 + PCDH24 EC1-4		T = 0, 60; R = 1, 2	4
	CDHR5 EC1-2 + PCDH24 EC1-4		T = 0, 60; R = 1, 2	4
	CDHR5 EC1-3 + PCDH24 EC1-4		T = 0, 60; R = 1, 2	4
	CDHR5 EC1-4 + PCDH24 EC1-4		T = 0, 60; R = 1, 2	5
	CDHR5 EC1-4 + PCDH24 EC1		T = 0, 60; R = 1, 2	3
	CDHR5 EC1-4 + PCDH24 EC1-2		T = 0, 60; R = 1, 2	3
	CDHR5 EC1-4 + PCDH24 EC1-3		T = 0, 60; R = 1, 2	3
	CDHR5 EC1-4 + PCDH24 EC1-MAD10		T = 0, 60; R = 1, 2	3
	CDHR5 EC1 + PCDH24 EC1-2		T = 0, 60; R = 1, 2	3
Mouse	CDHR5 EC1 + PCDH24 EC1-4		T = 0, 60; R = 1, 2	3
	CDHR5 EC1-2 + PCDH24 EC1-4		T = 0, 60; R = 1, 2	3
	CDHR5 EC1-3 + PCDH24 EC1-4		T = 0, 60; R = 1, 2	3
	CDHR5 EC1-4 + PCDH24 EC1-4		T = 0, 60; R = 1, 2	3
	CDHR5 EC1-4 + PCDH24 EC1		T = 0, 60; R = 1, 2	3
	CDHR5 EC1-4 + PCDH24 EC1-2		T = 0, 60; R = 1, 2	3
	CDHR5 EC1-4 + PCDH24 EC1-3		T = 0, 60; R = 1, 2	3
	CDHR5 EC1-4 + PCDH24 EC1-MAD10		T = 0, 60; R = 1, 2	3
	CDHR5 EC1-2 + PCDH24 EC1-2		T = 0, 60; R = 1, 2	3

* Used for the *mm* PCDH24 EC1-MAD10 as a control
