# Supplementary material for: Heterophilic and homophilic cadherin interactions in intestinal intermicrovillar links are species dependent
Source: PLoS Biol. 2021 Dec 6;19(12):e3001463. doi: 10.1371/journal.pbio.3001463 (PMC8691648; doi:10.1371/journal.pbio.3001463)
Supplement: S5 Table — (PDF) [file pbio.3001463.s025.pdf]

S5 Table. Accession numbers of PCDH24 sequences used in Consurf.

Accession Number	Species	Common Name
NP_001165447.1	*Homo sapiens*	Human
XP_014928203.2	*Acinonyx jubatus*	Cheetah
NP_001179233.2	*Bos taurus*	Cow
XP_005619238.1	*Canis lupus familiaris*	Dog
XP_022420063.1	*Delphinapterus leucas*	Beluga whale
XP_019693959.2	*Felis catus*	Domestic cat
NP_001028536.2	*Mus musculus*	Mouse
XP_004008751.3	*Ovis aries*	Sheep
XP_016809791.1	*Pan troglodytes*	Chimpanzee
XP_214434.4	*Rattus norvegicus*	Norwegian rat
XP_013850328.2	*Sus scrofa*	Pig
XP_014996966.2	*Macaca mulatta*	Rhesus macaque
XP_015307761.1	*Macaca fascicularis*	Crab-eating macaque
XP_012974778.1	*Mesocricetus auratus*	Golden hamster
XP_004737588.1	*Mustela putorius furo*	Domestic ferret
XP_023472938.1	*Equus caballus*	Horse
XP_029075003.1	*Monodon monoceros*	Narwhal
XP_028908976.1	*Ornithorhynchus anatinus*	Platypus
XP_008253668.2	*Oryctolagus cuniculus*	Rabbit
XP_008988594.1	*Callithrix jacchus*	White-tufted-ear marmoset
XP_024102684.1	*Pongo abelii*	Sumatran orangutan
XP_003473404.1	*Cavia porcellus*	Domestic guinea pig
XP_027263978.1	*Cricetulus griseus*	Chinese hamster
XP_023371131.1	*Otolemur garnettii*	Small-eared galago
XP_003806887.1	*Pan paniscus*	Pygmy chimpanzee
XP_003900612.1	*Papio anubis*	Olive baboon
XP_018883422.2	*Gorilla gorilla gorilla*	Western lowland gorilla
XP_004284878.1	*Orcinus orca*	Killer Whale
XP_019798890.1	*Tursiops truncatus*	Common bottlenose dolphin
XP_019329391.1	*Aptenodytes forsteri*	Emperor penguin
XP_019141545.2	*Corvus cornix cornix*	Crow
XP_015149366.1	*Gallus gallus*	Chicken
XP_027752218.1	*Empidonax traillii*	Willow Flycatcher
XP_027574630.1	*Pipra filicauda*	Wire-tailed manakin
XP_027538397.1	*Neopelma chrysocephalum*	Saffron-crested tyrant-manakin
XP_027499547.1	*Corapipo altera*	White-ruffed manakin
XP_026714386.1	*Athene cunicularia*	Burrowing owl
XP_025978389.1	*Dromaius novaehollandiae*	Emu
XP_025903300.1	*Nothoprocta perdicaria*	Chilean tinamou
XP_023791618.1	*Cyanistes caeruleus*	Blue tit
XP_021266449.1	*Numida meleagris*	Helmeted guineafowl
XP_017683677.1	*Lepidothrix coronata*	Blue-crowned manakin
XP_015731588.1	*Coturnix japonica*	Japanese quail

XP_015496956.1	*Parus major*	Great tit
XP_014803238.1	*Calidris pugnax*	Ruff
XP_013797945.1	*Apteryx australis mantelli*	North Island brown kiwi
XP_011569753.1	*Aquila chrysaetos canadensis*	American golden eagle
XP_019475977.1	*Meleagris gallopavo*	Turkey
XP_005053635.1	*Ficedula albicollis*	Collared flycatcher
XP_027634673.1	*Falco peregrinus*	Peregrine falcon
XP_027657949.1	*Falco cherrug*	Saker falcon
XP_026647227.1	*Zonotrichia albicollis*	White-throated sparrow
XP_021144536.1	*Columba livia*	Rock pigeon
XP_014108468.1	*Pseudopodoces humilis*	Tibetan ground-tit
XP_017583659.1	*Corvus brachyrhynchos*	American crow
XP_008939384.1	*Merops nubicus*	Carmine bee-eater
XP_009089922.2	*Serinus canaria*	Common canary
XP_009331800.1	*Pygoscelis adeliae*	Adelie penguin
XP_008119531.1	*Anolis carolinensis*	Green anole
XP_026576910.1	*Pseudonaja textilis*	Eastern brown snake
XP_026540941.1	*Notechis scutatus*	Mainland tiger snake
XP_020646672.1	*Pogona vitticeps*	Central bearded dragon
XP_019390868.1	*Crocodylus porosus*	Australian saltwater crocodile
XP_013925415.1	*Thamnophis sirtalis*	Common garter snake
XP_022532075.1	*Astyanax mexicanus*	Mexican tetra
XP_017214654.2	*Danio rerio*	Zebrafish
XP_021417551.1	*Oncorhynchus mykiss*	Rainbow trout
XP_014053985.1	*Salmo salar*	Atlantic salmon
XP_007904435.1	*Callorhinchus milii*	Elephant shark
XP_024139064.1	*Oryzias melastigma*	Indian Medaka
XP_022607721.1	*Seriola dumerili*	Greater amberjack
XP_015832622.1	*Nothobranchius furzeri*	Turquoise killifish
XP_004073569.1	*Oryzias latipes*	Japanese medaka
XP_029021466.1	*Betta splendens*	Siamese fighting fish
XP_028315581.1	*Gouania willdenowi*	Blunt-snouted clingfish
XP_028271476.1	*Parambassis ranga*	Indian glassy fish
XP_027865185.1	*Xiphophorus couchianus*	Monterrey platyfish
XP_026993994.1	*Tachysurus fulvidraco*	Yellow catfish
XP_026870202.1	*Electrophorus electricus*	Electric eel
XP_026802869.1	*Pangasianodon hypophthalmus*	Striped catfish
XP_026214145.1	*Anabas testudineus*	Climbing perch
XP_026186036.1	*Mastacembelus armatus*	Zig-zag eel
XP_026045273.1	*Astatotilapia calliptera*	Eastern happy
XP_019200886.1	*Oreochromis niloticus*	Nile tilapia
XP_014326181.1	*Xiphophorus maculatus*	Southern platyfish
XP_014187335.1	*Haplochromis burtoni*	Burton's mouthbrooder
XP_014342354.1	*Latimeria chalumnae*	Coelacanth

XP_007561596.1	*Poecilia formosa*	Amazon molly
XP_024918834.1	*Cynoglossus semilaevis*	Tongue sole
XP_010735796.2	*Larimichthys crocea*	Large yellow croaker
XP_010898483.1	*Esox lucius*	Northern pike
XP_012724017.1	*Fundulus heteroclitus*	Mummichog
XP_013870118.1	*Austrofundulus limnaeus*	Killifish
XP_014849149.1	*Poecilia mexicana*	Atlantic molly
