# Supplementary material for: Heterophilic and homophilic cadherin interactions in intestinal intermicrovillar links are species dependent
Source: PLoS Biol. 2021 Dec 6;19(12):e3001463. doi: 10.1371/journal.pbio.3001463 (PMC8691648; doi:10.1371/journal.pbio.3001463)
Supplement: S6 Table — (PDF) [file pbio.3001463.s026.pdf]

S6 Table. Accession numbers of CDHR5 sequences used in Consurf.

Accession Number	Species	Common Name
NP_068743.2	*Homo sapiens*	Human
NP_001107794.1	*Mus musculus*	Mouse
XP_027815739.1	*Ovis aries*	Sheep
XP_022421100.1	*Delphinapterus leucas*	Beluga whale
XP_020835078.1	*Phascolarctos cinereus*	Koala
XP_016775501.1	*Pan troglodytes*	Chimpanzee
XP_006230571.1	*Rattus norvegicus*	Norway rat
XP_013845324.2	*Sus scrofa*	Pig
NP_001096796.1	*Bos taurus*	Cow
XP_019668438.1	*Felis catus*	Domestic cat
XP_023510521.1	*Equus caballus*	Horse
XP_014437989.1	*Tupaia chinensis*	Chinese tree shrew
XP_012973076.1	*Mesocricetus auratus*	Golden hamster
XP_029064308.1	*Monodon monoceros*	Narwhal
XP_028642741.1	*Grammomys surdaster*	African woodland thicket rat
XP_028370928.1	*Phyllostomus discolor*	Pale spear-nosed bat
XP_025847484.1	*Vulpes vulpes*	Red fox
XP_025717837.1	*Callorhinus ursinus*	Northern ful seal
XP_024430321.1	*Desmodus rotundus*	Common vampire bat
XP_020767741.1	*Odocoileus virginianus texanus*	White-tailed deer
XP_009281978.1	*Aptenodytes forsteri*	Emporer penguin
XP_025966497.1	*Dromaius novaehollandiae*	Emu
XP_025928961.1	*Apteryx rowi*	Okarito brown kiwi
XP_025900649.1	*Nothoprocta perdicaria*	Chilean tinamou
XP_023784276.1	*Cyanistes caeruleus*	Blue tit
XP_015485075.1	*Parus major*	Great tit
XP_015718593.1	*Coturnix japonica*	Japanese quail
XP_014796130.1	*Calidris pugnax*	Ruff
XP_014734396.1	*Sturnus vulgaris*	Common starling
XP_013800822.1	*Apteryx australis mantelli*	North Island brown kiwi
XP_013053829.1	*Anser cygnoides domesticus*	Domestic goose
XP_011595784.1	*Aquila chrysaetos canadensis*	American golden eagle
XP_010709142.1	*Meleagris gallopavo*	Turkey
XP_010562687.1	*Haliaeetus leucocephalus*	Bald eagle
XP_010297449.1	*Balearica regulorum gibbericeps*	East African grey crowned-crane
XP_010292632.1	*Phaethon lepturus*	White-tailed tropicbird
XP_010225820.1	*Tinamus guttatus*	White-throated tinamou
XP_010207507.1	*Colius striatus*	Speckled mousebird
XP_007907548.1	*Callorhinchus milii*	Elephant shark
XP_021454527.1	*Oncorhynchus mykiss*	Rainbow trout
XP_021326278.1	*Danio rerio*	Zebrafish
XP_022525707.1	*Astyanax mexicanus*	Mexican tetra
XP_024120964.1	*Oryzias melastigma*	Indian medaka

XP_023807528.1	*Oryzias latipes*	Japanese medaka
XP_029312546.1	*Cottoperca gobio*	Channel bull blenny
XP_027008843.1	*Tachysurus fulvidraco*	Yellow catfish
XP_026168289.1	*Mastacembelus armatus*	Zig-zag eel
XP_019112627.2	*Larimichthys crocea*	Large yello croaker
XP_017341402.1	*Ictalurus punctatus*	Channel catfish
XP_016392038.1	*Sinocyclocheilus rhinocerous*	Rayfinned fish
XP_016324417.1	*Sinocyclocheilus anshuiensis*	Rayfinned fish
XP_015227070.1	*Cyprinodon variegatus*	Sheepshead minnow
XP_021163353.1	*Fundulus heteroclitus*	Mummichog
XP_012674652.1	*Clupea harengus*	Atlantic herring
XP_005938389.1	*Haplochromis burtoni*	Burton's mouthbrooder
XP_004556350.2	*Maylandia zebra*	Zebra mbuna
XP_008106729.1	*Anolis carolinensis*	Green anole
XP_019340716.1	*Alligator mississippiensis*	American alligator
XP_028591121.1	*Podarcis muralis*	Common wall lizard
XP_026562223.1	*Pseudonaja textilis*	Eastern brown snake
XP_026523924.1	*Notechis scutatus*	Mainland tiger snake
XP_026513403.1	*Terrapene carolina triunguis*	Three-toed box turtle
XP_019402850.1	*Crocodylus porosus*	Australian saltwater crocodile
XP_019375347.1	*Gavialis gangeticus*	Gharial
XP_015745583.1	*Python bivittatus*	Burmese python
XP_013925033.1	*Thamnophis sirtalis*	Common garter snake
XP_014429985.1	*Pelodiscus sinensis*	Chinese softshell turtle
XP_025054327.1	*Alligator sinensis*	Chinese alligator
XP_023967188.1	*Chrysemys picta bellii*	Western painted turtle
