# Supplementary material for: Heterophilic and homophilic cadherin interactions in intestinal intermicrovillar links are species dependent
Source: PLoS Biol. 2021 Dec 6;19(12):e3001463. doi: 10.1371/journal.pbio.3001463 (PMC8691648; doi:10.1371/journal.pbio.3001463)
Supplement: S7 Table — (PDF) [file pbio.3001463.s027.pdf]

S7 Table. Summary of simulations

Label	System	Type	Start	Time (ns)	Pulling speed (nm/ns)	Average force peak (pN) [d]	Size of system (# atoms)	Box dimensions (Å^3)
Sim1	*Mm* PCDH24 EC1-3	Equil[a]	-	99.1	-	-	227,299	171 × 112 × 111
Sim2a	*Mm* PCDH24 EC1-3	Equil[a]	-	11.1	-	-	195,074	337 × 71 × 80
Sim2b		SMD[b]	S2a	1	10	1905.5		
Sim2c		SMD[b]	S2a	10	1	1385.4		
Sim2d		SMD[b]	S2a	70	0.1	1216.0		
Sim3a	*Hs* PCDH24 EC1-2 I	Equil[a]	-	10.8	-	-	125,348	227 × 88 × 67
Sim3b		SMD[c]	S3a	1	10	675.2		
Sim3c		SMD[c]	S3a	21	1	383.9		
Sim3d		SMD[c]	S3a	58.6	0.1	229.2		
Sim4a	*Hs* PCDH24 EC1-2 II	Equil[a]	-	10.8	-	-	125,259	227 × 88 × 68
Sim4b		SMD[c]	S4a	1.25	10	1070.4		
Sim4c		SMD[c]	S4a	10	1	703.7		
Sim4d		SMD[c]	S4a	70	0.1	540.9		
Sim5	*Hs* PCDH24 EC1-2 / *Hs* CDHR5 EC1-2	Equil[a]	-	11.4	-	-	152,209	227 × 88 × 82

[a] Indicates simulations that consisted of 1,000 steps of minimization, 100 ps of dynamics with the protein backbone constrained, 1 ns of free dynamics in the *NpT* ensemble (γ = 1 ps^{-1}), and then of free dynamics in the *NpT* ensemble (γ = 0.1 ps^{-1}) for the rest of the time.

[b] Indicates SMD simulations in which the force was applied to the centers of mass (COM) of EC1 (p.N1 to p.E95) and EC3 (p.P215 and p.T324).

[c] Indicates SMD simulations in which the force was applied to the Cα atoms of residues p.P215 of chains A and D.

[d] Average peak forces were calculated from peak forces applied to stretched Cα atoms or COM at each end and using 50-ps running averages.
