# Supplementary material for: Heterophilic and homophilic cadherin interactions in intestinal intermicrovillar links are species dependent
Source: PLoS Biol. 2021 Dec 6;19(12):e3001463. doi: 10.1371/journal.pbio.3001463 (PMC8691648; doi:10.1371/journal.pbio.3001463)

The following two images are the same blot. The first image is a white light image of the prestained ladder (Lanes 1 and 10) which were used as a guide for all westerns and the second image is the chemiluminescent image. The prestained ladder has two markers (80 kDa and 30 kDa) that show in the chemiluminescent image. Lanes 2-9 of the chemiluminescent image were used in Fig 3M (flipped horizontally in the figure panel). The lanes are listed as follows:

Lane 1 – iBright Prestained ladder (MW markers in kDa)
Lane 2 – *hs* PCDH24 EC1-10Fc
Lane 3 – *hs* PCDH24 EC1-7Fc
Lane 4 – *hs* PCDH24 EC1-6Fc
Lane 5 – *hs* PCDH24 EC1-5Fc
Lane 6 – *hs* PCDH24 EC1-4Fc
Lane 7 – *hs* PCDH24 EC1-3Fc
Lane 8 – *hs* PCDH24 EC1-2Fc
Lane 9 – *hs* PCDH24 EC1Fc
Lane 10 – iBright Prestained ladder

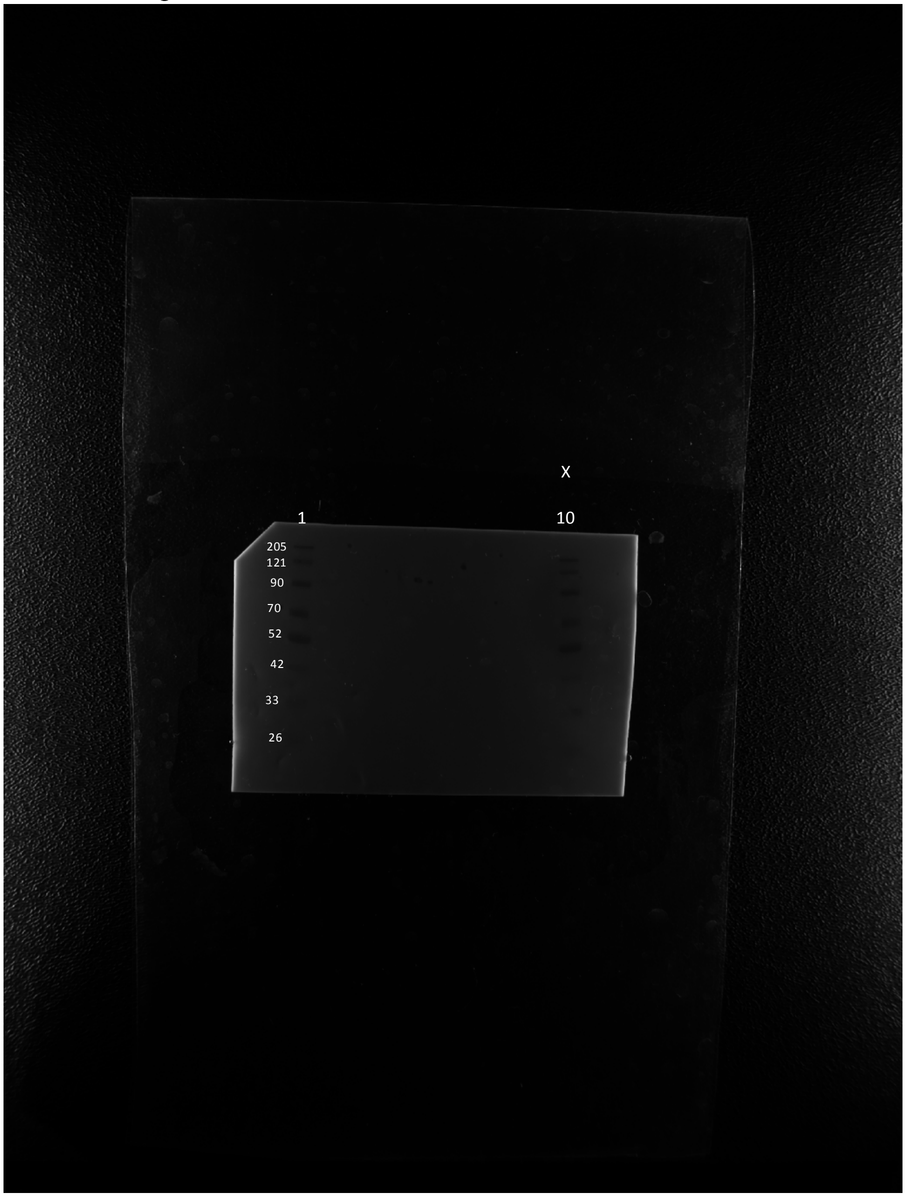

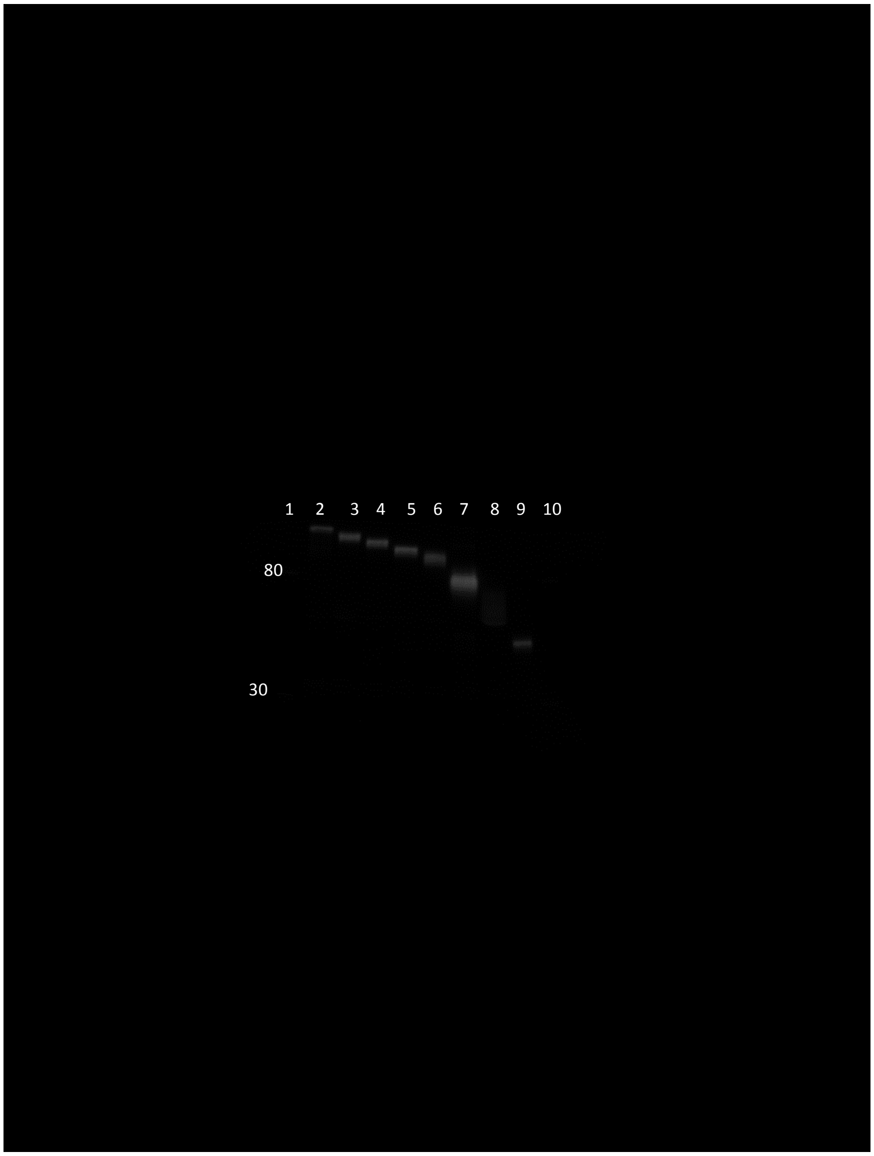
1 2 3 4 5 6 7 8 9 10
80
30

This image is the chemiluminescent image for the blot shown in Fig 4J for *hs* PCDH24 EC1-3Fc and *hs* PCDH24 EC1-2Fc mutants. The prestained ladder has two markers (80 kDa and 30 kDa) that show in the chemiluminescent image. The lanes are listed as follows:

Lane 1 – *hs* PCDH24 EC1-2Fc
Lane 2 – *hs* PCDH24 EC1-2Fc Y67A
Lane 3 – *hs* PCDH24 EC1-2Fc Y71A
Lane 4 – iBright Prestained ladder (MW markers in kDa)
Lane 5 – *hs* PCDH24 EC1-3Fc
Lane 6 – *hs* PCDH24 EC1-3Fc Y67A
Lane 7 – *hs* PCDH24 EC1-3Fc Y71A

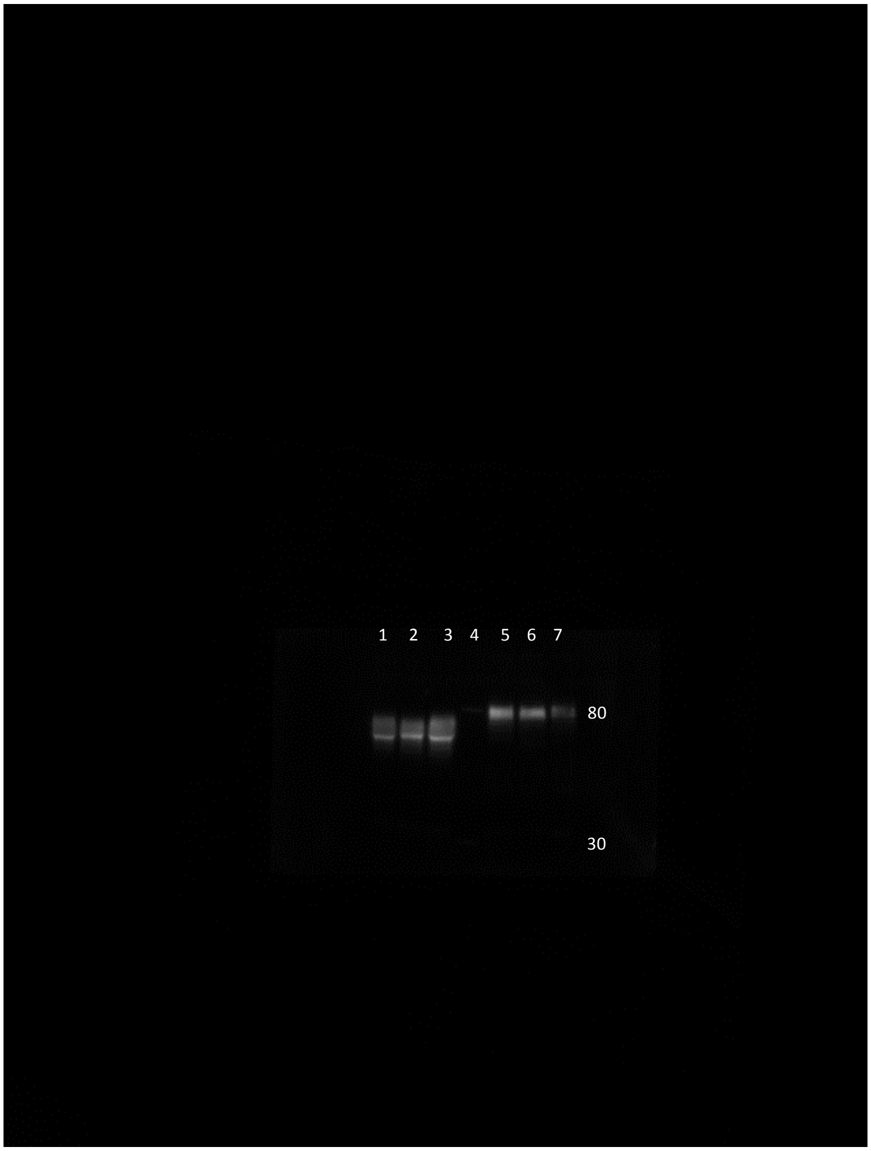

This image is the chemiluminescent image for the blot shown in Fig 5G for *hs* PCDH24 EC1-10Fc (Lane 2) and *mm* PCDH24 EC1-10Fc (Lane 1). The prestained ladder has two markers (80 kDa and 30 kDa) that show in the chemiluminescent image. The lanes are listed as follows:

Lane 1 – *mm* PCDH24 EC1-10Fc
Lane 2 – *hs* PCDH24 EC1-10Fc
Lane 3 – iBright Prestained ladder (MW markers in kDa)
Lane 5 – 10 - X

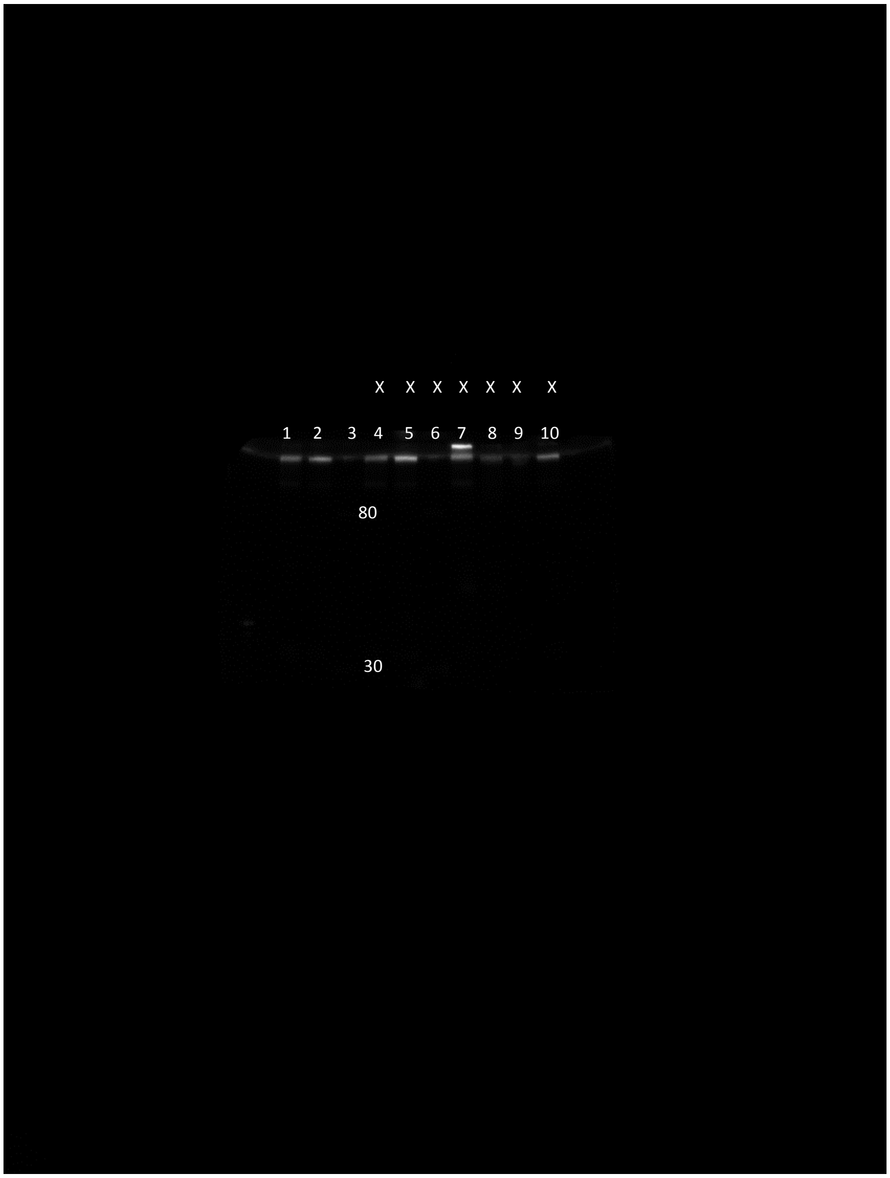

This image is the chemiluminescent image for the blot shown in Fig 5G for *hs* CDHR5 EC1-4Fc (Lane 3). The prestained ladder has two markers (80 kDa and 30 kDa) that show in the chemiluminescent image. The lanes are listed as follows:
Lane 1, 2 – X
Lane 3 – *hs* CDHR5 EC1-4Fc
Lane 4 – iBright Prestained ladder (MW markers in kDa)

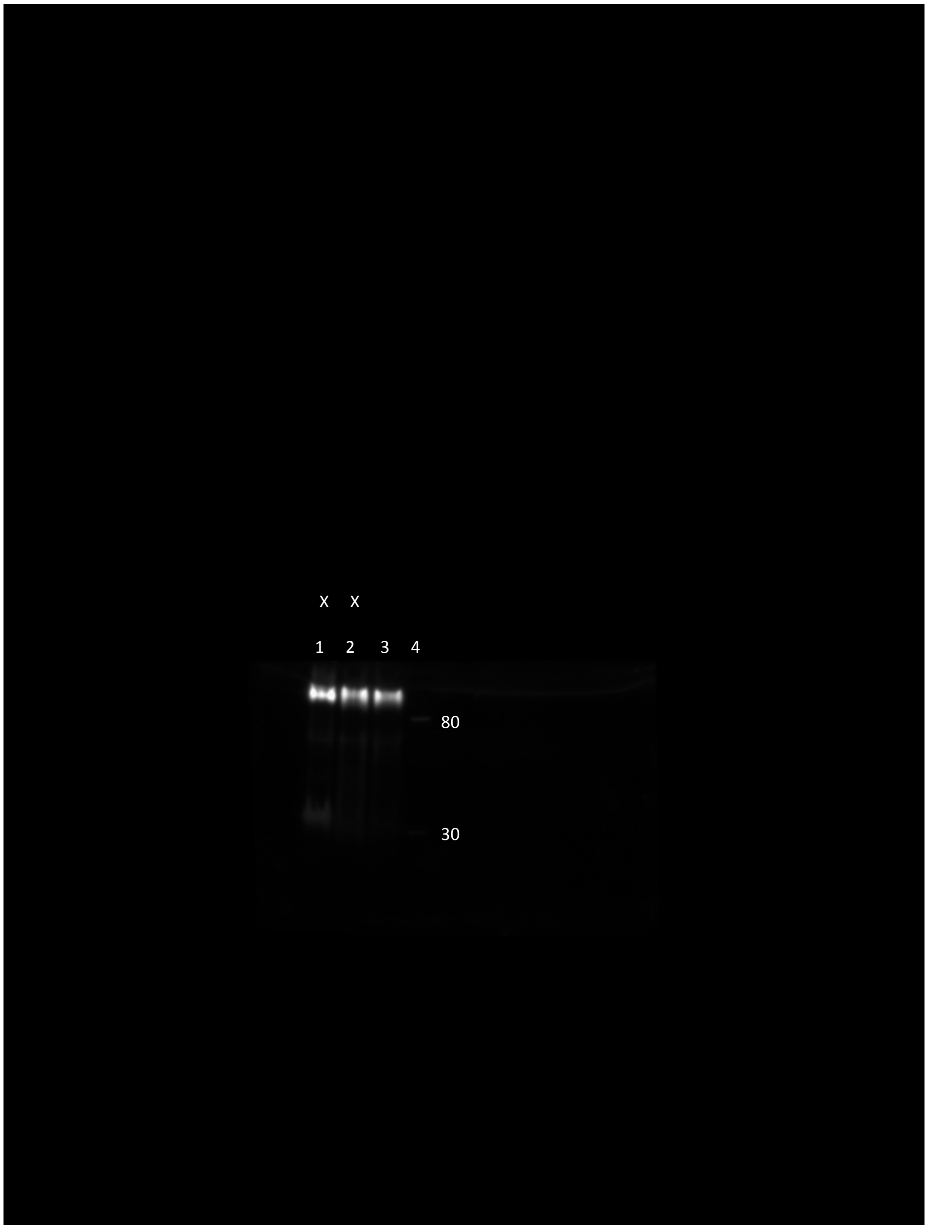

This image is the chemiluminescent image for the blot shown in Fig 5G for *mm* CDHR5 EC1-4Fc (Lane 7). The prestained ladder has two markers (80 kDa and 30 kDa) that show in the chemiluminescent image. The lanes are listed as follows:

Lane 1-5, 8-11 – X

Lane 6 – iBright Prestained ladder (MW markers in kDa)

Lane 7 – *mm* CDHR5 EC1-4Fc

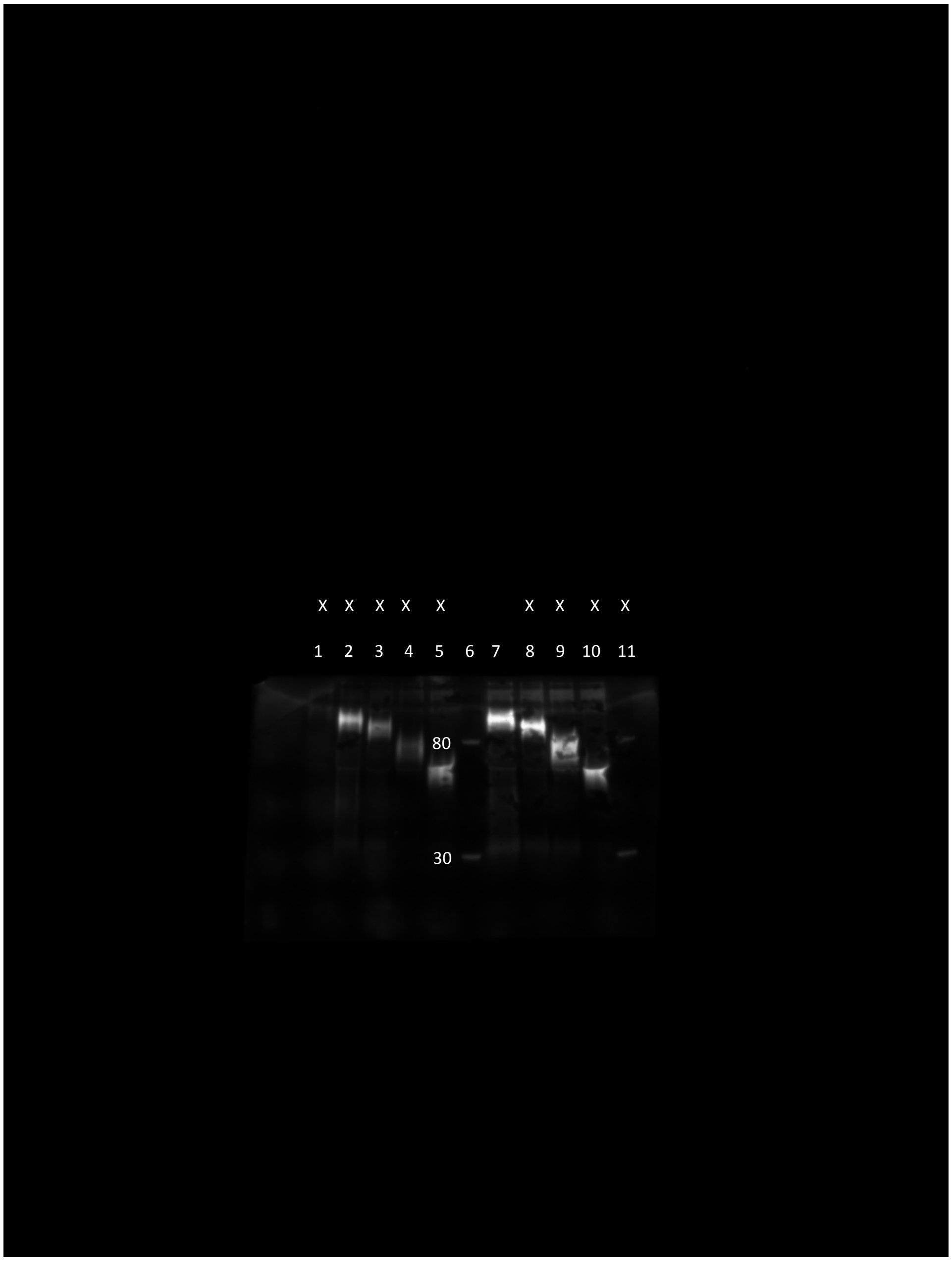

This image is the chemiluminescent image for the blot shown in Fig 6I (flipped horizontally in the figure panel). The prestained ladder has two markers (80 kDa and 30 kDa) that show in the chemiluminescent image. Lanes 2-6 of the chemiluminescent image were used in Fig 6I. The lanes are listed as follows:

Lane 1 – X
Lane 2 – *mm* CDHR5 EC1-4Fc
Lane 3 – *mm* CDHR5 EC1-3Fc
Lane 4 – *mm* CDHR5 EC1-2Fc
Lane 5 – *mm* CDHR5 EC1Fc
Lane 6 – iBright Prestained ladder (MW markers in kDa)
Lane 7-11 – X

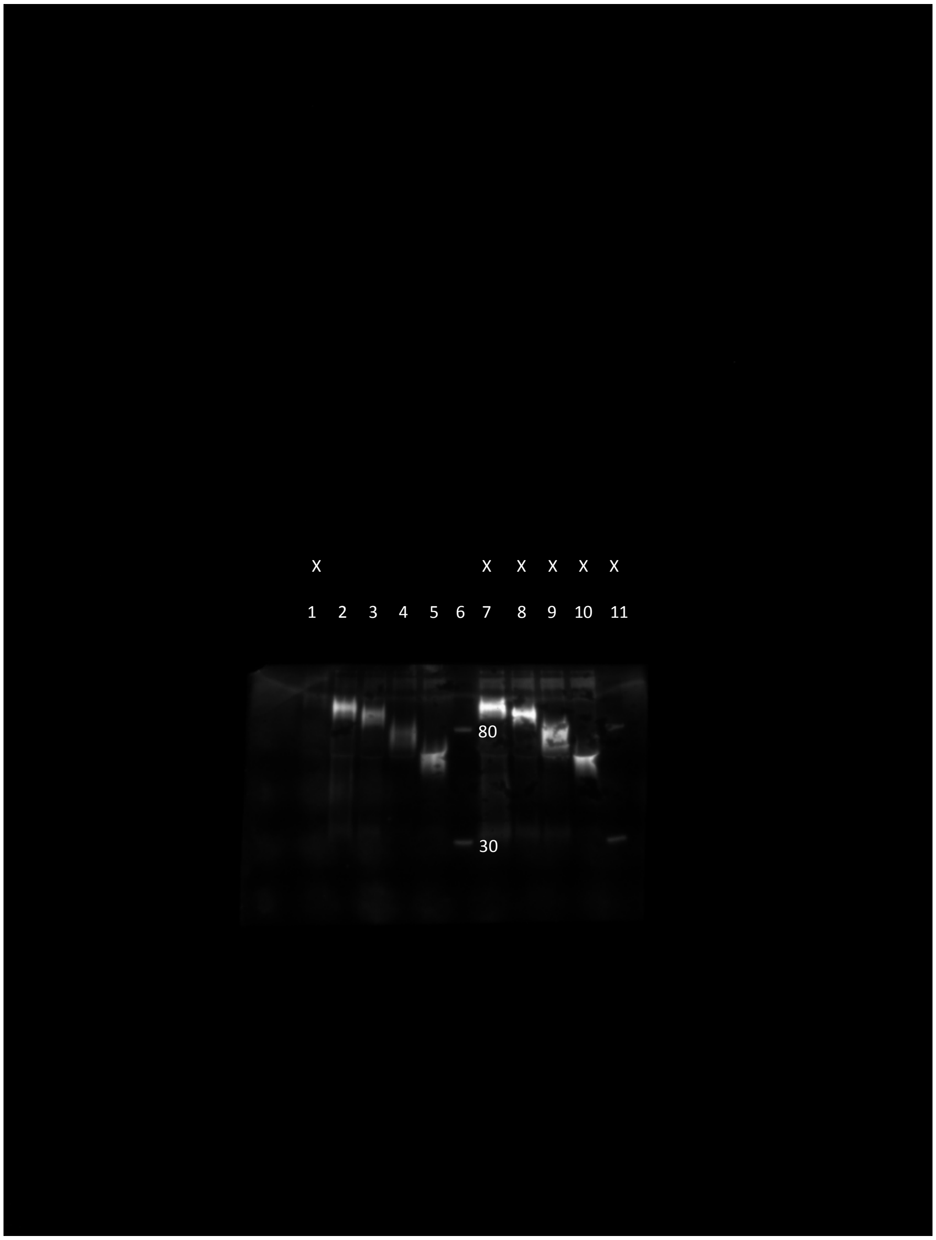

This image is the chemiluminescent image for the blot shown in Fig 6I for *mm* CDHR5 EC1-4Fc mutants R82G and E84G (Lane 4-5). The prestained ladder has two markers (80 kDa and 30 kDa) that show in the chemiluminescent image. The lanes are listed as follows:

Lane 1 – iBright Prestained ladder (MW markers in kDa)
Lane 2, 3 – X
Lane 4 – *mm* CDHR5 EC1-4Fc R82G
Lane 5 – *mm* CDHR5 EC1-4Fc E84G
Lane 6-12 – X

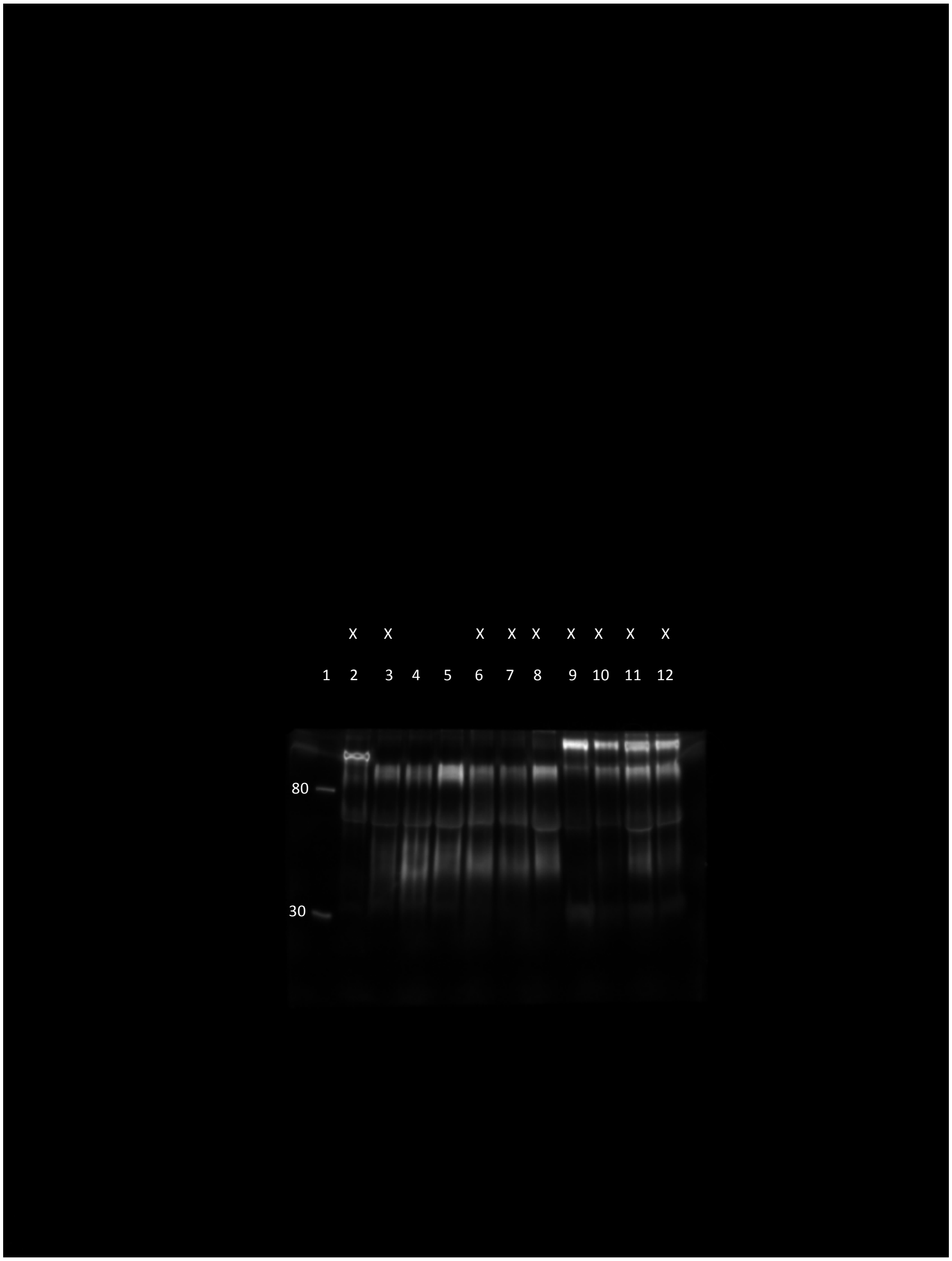

This image is the chemiluminescent image for the blot shown in Fig 7K. The prestained ladder has two markers (80 kDa and 30 kDa) that show in the chemiluminescent image. Lanes 1-10 of the chemiluminescent image were used in Fig 7K. The lanes are listed as follows:
Lane 1 – *hs* PCDH24 EC1Fc
Lane 2 – *hs* PCDH24 EC1-2Fc
Lane 3 – *hs* PCDH24 EC1-3Fc
Lane 4 – *hs* PCDH24 EC1-4Fc
Lane 5 – *hs* PCDH24 EC1-10Fc
Lane 6 – *hs* CDHR5 EC1Fc
Lane 7 – *hs* CDHR5 EC1-2Fc
Lane 8 – *hs* CDHR5 EC1-3Fc
Lane 9 – *hs* CDHR5 EC1-4Fc
Lane 10 – iBright Prestained ladder (MW markers in kDa)

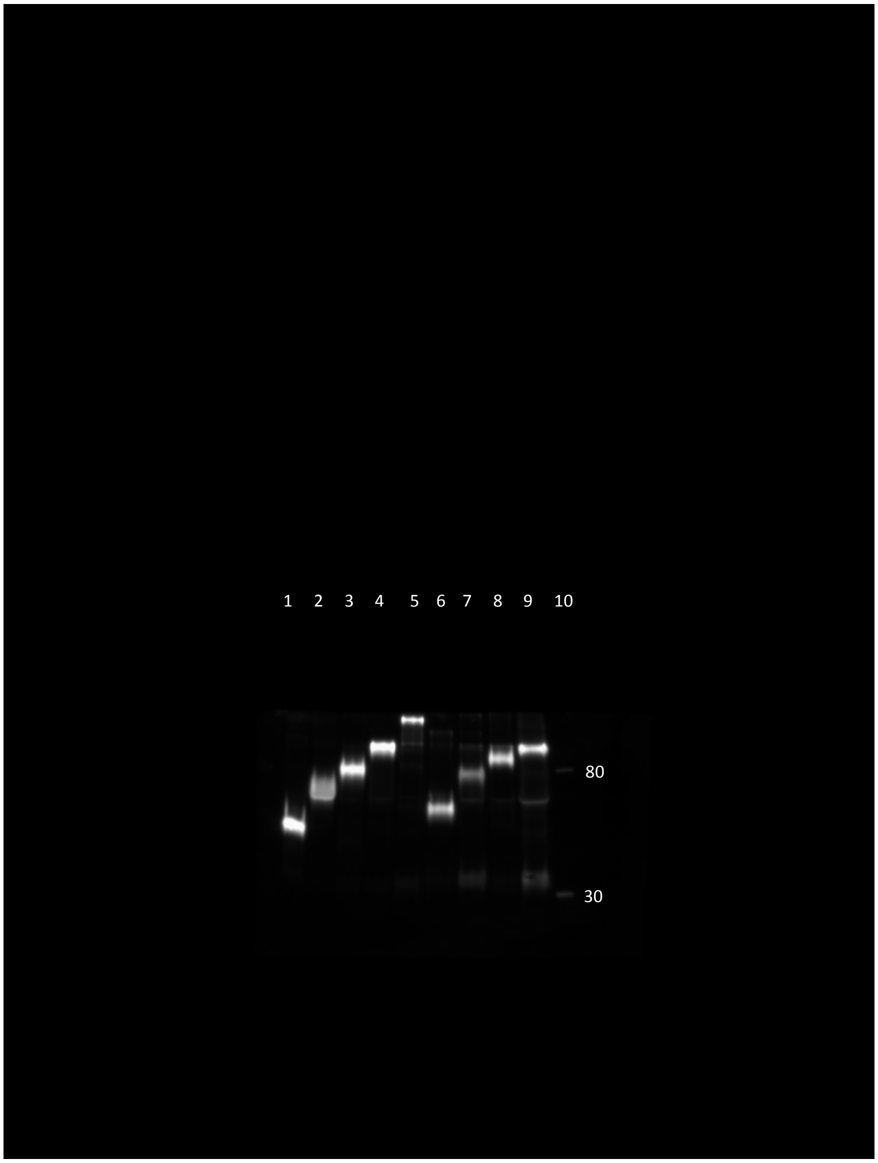

This image is the chemiluminescent image for the blot shown in Fig 7K. The prestained ladder has two markers (80 kDa and 30 kDa) that show in the chemiluminescent image. Lanes 1-10 of the chemiluminescent image were used in Fig 7K. The lanes are listed as follows:

Lane 1 – iBright Prestained ladder (MW markers in kDa)
Lane 2 – *mm* PCDH24 EC1Fc
Lane 3 – *mm* PCDH24 EC1-2Fc
Lane 4 – *mm* PCDH24 EC1-3Fc
Lane 5 – *mm* PCDH24 EC1-4Fc
Lane 6 – *mm* PCDH24 EC1-10Fc
Lane 7 – *mm* CDHR5 EC1Fc
Lane 8 – *mm* CDHR5 EC1-2Fc
Lane 9 – *mm* CDHR5 EC1-3Fc
Lane 10 – *mm* CDHR5 EC1-4Fc
Lane 11 – iBright Prestained ladder (MW markers in kDa)

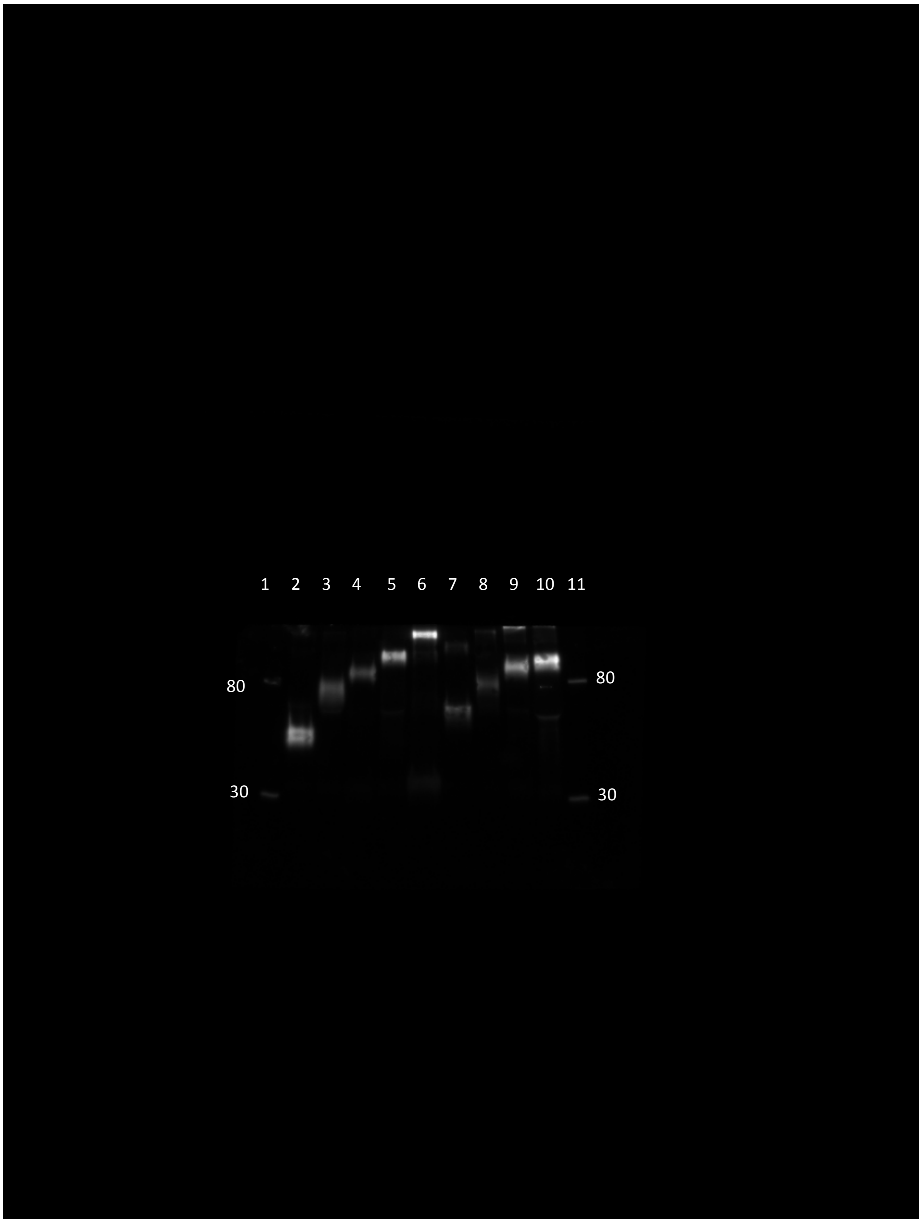

Supplement: S1 Raw Images — This pdf file contains original gel images used to generate Figs 3M, 4J, 5G, 6I and 7K. (PDF) [file pbio.3001463.s041.pdf]
