# Supplementary material for: Heterophilic and homophilic cadherin interactions in intestinal intermicrovillar links are species dependent
Source: PLoS Biol. 2021 Dec 6;19(12):e3001463. doi: 10.1371/journal.pbio.3001463 (PMC8691648; doi:10.1371/journal.pbio.3001463)
Supplement: S5 Fig — All 9 EC repeats for each species are aligned to each other (EC1 to EC9). Conserved calcium-binding motifs are labeled on top of the alignment. EC, extracellular cadherin; PCDH24, protocadherin-24. (PDF) [file pbio.3001463.s005.pdf]

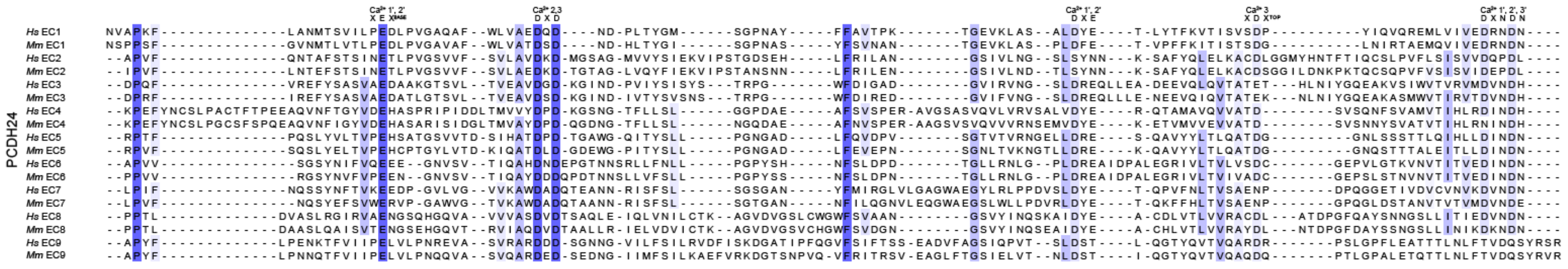

S5 Fig. Sequence alignment of *hs* and *mm* PCDH24 EC repeats. All 9 EC repeats for each species are aligned to each other (EC1 to EC9). Conserved calcium-binding motifs are labeled on top of the alignment.
