# Supplementary material for: Heterophilic and homophilic cadherin interactions in intestinal intermicrovillar links are species dependent
Source: PLoS Biol. 2021 Dec 6;19(12):e3001463. doi: 10.1371/journal.pbio.3001463 (PMC8691648; doi:10.1371/journal.pbio.3001463)
Supplement: S15 Fig — All 4 EC repeats for each species are aligned to each other (EC1 to EC4). CDHR5, cadherin-related family member 5; EC, extracellular cadherin. (PDF) [file pbio.3001463.s015.pdf]

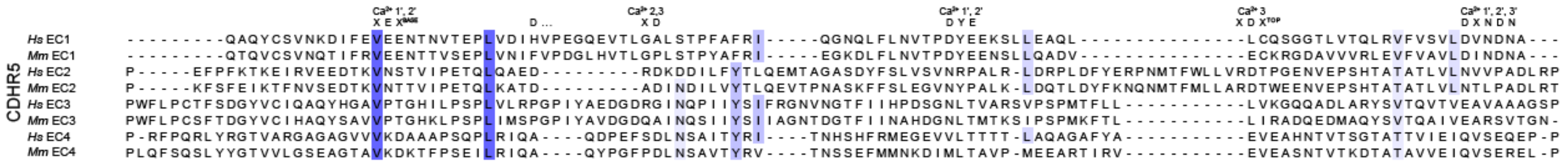

S15 Fig. Sequence alignment of *hs* and *mm* CDHR5 EC repeats. All 4 EC repeats for each species are aligned to each other (EC1 to EC4).
