# Supplementary material for: Heterophilic and homophilic cadherin interactions in intestinal intermicrovillar links are species dependent
Source: PLoS Biol. 2021 Dec 6;19(12):e3001463. doi: 10.1371/journal.pbio.3001463 (PMC8691648; doi:10.1371/journal.pbio.3001463)
Supplement: Attachment — Submitted filename: ResponseReviewers-V6.pdf [file pbio.3001463.s042.pdf]

We thank all three reviewers for their thorough and thoughtful comments. Following their suggestions we have conducted additional experiments to quantify binding between PCDH24 and CDHR5 and to provide more details about interfaces that may mediate their homophilic and heterophilic adhesion. New results reported in our revised manuscript include a crystal structure (*hs* PCDH24 EC1-2 form II, PDB code 7N86) that revealed a bona fide interface for homophilic adhesion mediated by *hs* PCDH24, bead aggregation experiments with protein fragments carrying mutations inspired by the structure that test this interface, as well as surface plasmon resonance (SPR) experiments that characterize the mouse heterophilic complex. These new data provide a confirmation of the *trans* homodimer that human PCDH24 is capable of forming in the absence of CDHR5 and a more quantitative characterization of the minimal adhesive unit in the mouse heterophilic complex. These new findings have been incorporated into our revised manuscript along with edits that help clarify and address reviewers' requests and concerns.

A detailed response follows, including reviewer comments in *italics*. Modifications in the manuscript text are marked in the manuscript.

Reviewers' comments

Rev. 1:
In their study, Gray et al. present a crystal structure of the extracellular cadherin repeats (EC) 1-2 of human protocadherin-24 (EC1-3 for the mouse protein) and EC domain 1-2 of the human Protocadherin Cadherin-related family member 5 (CDHR5) at very high resolution (2.3Å, 2.1Å and 1.9Å respectively). The heterophilic interaction between these two protocadherins has previously been reported to be essentially for microvilli development and function. Notably, the authors reveal a rather low sequence conservation of both proteins (in contrast to similar adhesion molecules of the classical cadherin and protocadherin family) and important structural differences between mouse and human proteins.

As a functional readout, the authors then used aggregation assays to demonstrate that human PCDH24 forms calcium-dependent self-aggregates (in trans) when equipped with a minimum of three EC repeats. In line with the structural differences described in this study, the extracellular domain of mouse PCDH24 does not aggregate. Notably, CDHR5 behaves the other way around: Mouse CDHR5 self-aggregates, whereas PCDH24 does not. Testing the important heterophilic aggregation between PCDH24 and CDHR5, the authors reveal that proteins of both species are capable of forming calcium-dependent hetero-aggregates.

The manuscript is well written and addresses an issue which is of high relevance for our understanding of microvilli development and function. The presented data are robust and well described. However, some important issues remain unclear and should be addressed to provide a complete picture of the molecular mechanism of the PCDH24-CDH5 module.

We thank reviewer 1 for their positive evaluation of the data presented in our manuscript.

Major points:
1. The calculation of the average identity across species should be better explained in the text and in the figure legend.

We have modified the Results section in the main text to make sure that the calculations are clear (page 5, lines 7-8 and page 5 line 23), with additional explanations in the Methods section (page 24, lines 11-12).

2. Page 17, line 9-10 (and discussion): The authors cannot conclude that PCDH24 EC1

exhibits a calcium-independent aggregation, because they argue some lines above that the calcium-independent aggregation of EC1 might be explained by a stabilization of the Fc Tag. This discrepancy should be tested in a different experimental setup.

The reviewer is right. We swapped the EC1 repeats of human and mouse PCDH24 to test this using bead aggregation assays. We do see calcium-dependent bead aggregation when human EC1 is attached to mouse PCDH24 EC2-MAD10 (data not shown). We have updated the results and conclusions to de-emphasize the possibility of calcium-independent bead aggregation mediated by human PCDH24 EC1, only highlighting that the Fc tag provides some stability to the EC1 construct alone (page 10, line 23).

3. Some important questions remain and should be addressed experimentally:
a. Is homophilic aggregation of human PCDH24 (and mouse CDHR5) a prerequisite for the formation of hetero-aggregates?

We have added a figure at the end of this response that includes results from a time lapse of the PCDH24 and CDHR5 heterophilic bead aggregation assays for the minimum adhesive units (both species), for the full-length mouse proteins, and for one set of protein fragments that does not aggregate. Results recorded at the T0 time point, immediately after the two sets of fluorescent beads are mixed, do not show homophilic *trans* aggregation of either *hs* PCDH24 or *mm* CDHR5. We only observed mixed heterophilic aggregates after 60 min during which time the setup was still but beads experienced Brownian motion. The lack of *trans* homophilic aggregation prior to heterophilic aggregation suggests that the *trans* homophilic interactions observed for *hs* PCDH24 and *mm* CDHR5 are not a prerequisite for *trans* heterophilic adhesion. Additionally, given that the mouse EC1-2 repeats are sufficient for *trans* heterophilic adhesion, but *mm* CDHR5 requires EC1-3 to mediate *trans* homophilic adhesion, it is unlikely that the mouse *trans* heterophilic complex requires a *trans* homophilic pre-cursor to occur. A short discussion on this topic has been added in page 17, lines 4 to 7.

b. Does cis-homo-dimerization occur in case of human PCDH24 and/or mouse CDHR5?

Unfortunately we do not have data that can answer this question, as now noted in page 17, lines 8 to 9. We agree that this is an important issue, and MAD10 in PCDH24 might be facilitating *cis* homo-dimerization, just as MAD12 does in PCDH15. However, we believe that properly identifying and characterizing *cis* interactions is beyond the scope of the present work, which is focused on the species-dependent *trans* bond.

c. Is the (cis or trans) homo-dimerization of one of the adhesion partners essential for trans-heterophilic aggregation?

Our bead aggregation assay data indicates that the mouse protein fragments do not require *trans* homophilic interactions for the heterophilic interaction to occur, since the heterophilic interaction is mediated by EC1-2 of both proteins, but *mm* CDHR5 requires EC1-3 for homophilic adhesion to occur. We have included a short comment on this topic in page 17, lines 6 to 7.

d. Is trans-heterophilic aggregation stronger than trans-homophilic interaction/are there binding preferences for either homo- or heterophilic interactions?

When we began testing the heterophilic interaction we did not know about the *trans* homodimerization of mouse CDHR5 (only human PCDH24 was known to form *trans* homodimers). We observed the mouse CDHR5 homophilic interaction when we removed EC2 from mouse PCDH24 (and this can be observed in Figures 7F and S18F where large red aggregates formed). Based on this initial observation, it seems that the mouse CDHR5 protein fragments prefer to bind to PCDH24 unless PCDH24 is absent or is missing one of the necessary EC repeats for *trans* interactions. Our new surface plasmon resonance (SPR) data provide confirmation of the heterophilic interaction with an observed K_D of ~16 μM (Figure S19) however, we were unable to obtain clear information about the homophilic interaction of CDHR5 with SPR. From the binding assays it can be concluded that the proteins prefer the heterophilic interaction over the species specific homophilic interactions, but because we do not have quantitative information on the homophilic interaction, it is difficult to say which binds stronger. We have included a short discussion on this topic in page 17 (lines 1 to 10).

Minor points:
Introduction: page 10, line 10. Some diseases should be given as examples (with more recent citations)

We have updated the introduction text to give disease examples with more recent citations (page 3, lines 9 to 10; references 18 to 20)

Page 10, line 15: The abbreviation CDHR should be introduced at first use

The CDHR abbreviation has been defined as “cadherin-related family member” in the introduction (page 3, line 13).

Page 12, line 5: PCDH24, CDHR5 and PCDH15 are not (classical) "cadherins"

These proteins have now been described as non-classical cadherins in the text (page 4, line 25).

Rev. 2:
This is a well-performed study that combines X-ray crystallography and bead aggregation assays to present the structural basis for the species dependent adhesive properties of PCDH24 and CDHR5, two non-classical cadherins that function in maintaining the intermicrovillar links in the small intestine. The authors determined the crystal structures of select N-terminal fragments from PCDH24 and CDHR5. By combining the structural observations with bead aggregation analysis, the authors report that homophilic and heterophilic interactions by PCDH24 and CDHR5 are species dependent involving distinct minimal adhesive units in different species. Overall, this work is of high quality and the results are significant. The data presented in this work will be of interest to readers studying cell adhesion molecules and in particular those interested in the molecular diversity of cadherins.

We appreciate the reviewer’s comments and share their enthusiasm about the data presented in our manuscript.

My main criticisms are:
1. Lack of solution biophysical characterization to validate the proposed adhesive models.

Bead aggregation is a reasonably good proxy to cell aggregation behavior, but rigorous solution biophysical methods such as analytical ultracentrifugation or isothermal titration calorimetry will be needed to corroborate the molecular interaction mechanisms proposed.

We understand that the bead aggregation assay is a “simple” semi-quantitative method for determining binding properties and the minimal adhesive unit of interacting proteins. We note, however, that analytical ultracentrifugation and isothermal titration calorimetry require prohibitively large amounts of proteins, and that these experiments do not probe the right configurations as proteins are not immobilized at their C-terminal ends as it occurs *in vivo* where transmembrane domains anchor them to the cell. We decided to try out surface plasmon resonance (SPR) experiments instead, and characterized the minimal mouse heterophilic complex obtaining a K_D of ~16 μM for the *mm* PCDH24 EC1-2Fc / *mm* CDHR5 EC1-2 interaction (Figure S19). A short discussion on this topic has been added in page 17 (lines 1 to 10).

2. Lack of mutagenesis studies to test the putative biological interfaces suggested by crystal packing interactions that bury meaningful amount of surface areas.

None of the crystal contacts observed in our original structures showed interfaces that could be successfully tested with our *in vitro* assays, hence our schematic diagram in the last figure of the original submission that highlighted specific EC repeats, but not specific interactions. We obtained a new crystal structure of *hs* PCDH24 EC1-2 at 3.2 Å resolution that shows a different arrangement of monomers in the asymmetric unit (*hs* PCDH24 EC1-2 form II, PDB 7N86; Table 1). Steered molecular dynamics simulations suggested that the largest antiparallel *trans interface* in this new structure is mechanically strong (new Figure S9C). In parallel, we tested several mutations inspired by the most likely *trans* homophilic interfaces from both crystal structures (forms I and II) and discovered two mutations that altered bead aggregation assay outcomes (new Figure 4). These mutations, p.Y67A and p.Y71A, modified residues at a large *trans* interface in the new *hs* PCDH24 EC1-2 II structure (Figure S10). When the single point mutation Y71A was introduced we observed an absence and a decrease of bead aggregation in assays carried out with *hs* PCDH24 EC1-2Fc Y71A and EC1-3Fc Y71A, respectively (new Figure 4). In contrast, the Y67A caused a notable increase in bead aggregation for *hs* PCDH24 EC1-2Fc Y67A and EC1-3Fc Y67A (new Figure 4). These new data strongly suggest that both residues are at a “bona fide” *hs* PCDH24 *trans* interface (new Figure 8B) mediating homophilic adhesion. We also speculate that this interface may serve as a good template for the heterophilic complexes (Figure S20), but believe that further tests are beyond the scope of the current manuscript.

Some specific comments:

1. Page 5, 2nd paragraph and Figure 1: "average sequence identity is 51.6% for CDH23 EC repeats and 45.5% for PCDH15 repeats……In contrast, average percent identity is 16.4% for PCDH24 and 11% for CDHR5, considerably lower when compared to values for CDH23 and PCDH15." I gather that the main point the authors want to make here is that the sequence identities for CDH23 and PCDH15 are higher than those for PCDH24 and CDHR5. But to make meaningfully quantitative comparisons between these two pairs using these numbers (with three or four significant digits), the exact same set of species (preferably from as diverse phyla as possible), needs to be selected for the alignment of each cadherin. But from Tables S2-S5, it seems that the set of species selected is not completely identical for each of these four

cadherins. For these comparisons, it would be better to report a range of numbers rather than specific average numbers.

We did intend to emphasize that CDH23 and PCDH15 sequences are overall more conserved than PCDH24 and CDHR5 sequences and understand the concern about using different sets of species in protein sequence alignments. We have therefore redone the individual EC alignments using the exact same set of sequences from 13 species for PCDH24, CDHR5, CDH23, and PCDH15. Figures and tables have been updated accordingly (Figure 1, S1, and S2 and Tables S1 and S2). We were able to obtain a variety of different species in mammals, birds, reptiles, and fish, as well as one amphibian. We have updated the text to include the new values, ranges, and averages obtained from these alignments.

2. Page 7, line 7: (S187:S201) needs to be changed to (C187:C201).

Thank you for catching this typo, which has been fixed (page 7, line 3).

3. Page 7, lines 23-24: "equilibrium and steered molecular dynamics (SMD) simulations of mm PCDH24 EC1-3 show that rigidity and mechanical strength are not compromised in this non-canonical linker." The simulations suggested that this linker is rigid, but what are the possible implications of this rigidity? This claim needs to be discussed further in detail or removed from the manuscript if it has little to do with the adhesive interaction mechanisms proposed in this work.

This is a good point and a short sentence commenting on the importance of rigidity for adhesion has been added in page 7, lines 21 to 22.

4. Page 8, 1st paragraph: "The sequence motifs involved in calcium coordination at site 0 are conserved, suggesting that crystallization conditions, including 3M LiCl, facilitated the opening of this site." Possible, but not certain without further experimental evidence, so I would use "may have facilitated" or something like that. Lithium chloride may or may not have facilitated the opening of this particular site. But if, as stated in this sentence, 3M lithium chloride in the crystallization solution disrupted calcium binding at site 0, which is clearly not a physiologically relevant condition, no functional implications can be deduced from the structures observed under this condition. Thus, it raises a question about the proposed cis interaction mechanism for mouse PCDH24 by N-terminal interlocking.

We have changed the text as suggested (page 8, line 3).

5. Page 8, the section of crystal contacts: as mentioned above in my main criticisms, a number of crystal packing interactions with reasonable buried surface areas were discussed in detail, but all just discussions with no experimental validations. Testing mutants designed to disrupt these interfaces would make this a much stronger paper.

We agree that mutants disrupting crystal contacts and impairing bead aggregation would provide evidence of the physiological relevance of these potential interfaces mediating adhesion. As mentioned above, none of the crystal contacts observed in our original structures showed interfaces that could be successfully tested with our *in vitro* assays. However, we did obtain a new crystal structure, *hs* PCDH24 EC1-2 II that shows a bona fide interface. We also identified two mutations that altered bead aggregation assays outcomes (p.Y67A and p.Y71A). These new data have been incorporated to the revised manuscript.

6. Page 9, lines 13-14: why use (p.N161) and (p.N281) instead of just (N161) and (N281) when referring to these glycosylation sites? The same comment applies to page 12, lines 6-7 where glycosylation sites are mentioned.

In other work we have been asked to differentiate between DNA coding sequence and protein amino acids by adding “c.” and “p.”, respectively. We have done this systematically in the text but will adapt to the journal format and editor’s preference.

Rev. 3:

This manuscript by Gray et al., tries to identify the structural determinants of the interactions between protocadherin-24 (PCDH24) and CDHR5. PCDH24 and CDHR5 are non-classical cadherins and their interactions drive the assembly of the intestinal brush border assembly. The authors present two x-ray crystal structures of PCDH24 (one from mouse and anther human) in addition to a human CDHR5 structure, all in their monomeric forms. These structures confirm similarities between PCDH24 to CDH23 and between CADHR5 and PCDH15. Several crystal contacts are described and it is suggested that these may facilitate the adhesion interfaces. In addition, the authors present bead aggregation assay to characterize the protein fragments that are required for adhesion. Using the aggregation assays with both the human and mouse proteins the authors suggest that these proteins exhibit species-dependent homophilic interactions and species-distinct heterophilic adhesive units. The technical aspects in this paper are well executed and are described in sufficient details.

Thank you for providing a positive assessment of the technical aspects of our work and its description.

My major criticism lies in the fact that it does not appear that the findings and conclusions of this current manuscript contribute to major advances in this field. In the absence of structures that include the adhesive interfaces, the physiological relevance of the crystal contacts presented here remain unclear. This criticism holds true for both heterophilic and homophilic interactions. Ultimately the reader does not obtain an answer regarding the structural determinants of the interactions between PCDH24 and CADH24. In the absence of structure that describe the interface I cannot recommend to accept this paper to Plos biology.

The main goals of our original submission were to report on the first PCDH24 and CDHR5 structures, to describe our characterization of the minimal subunits of adhesion for these proteins, and to report on the discovery of their species-dependent interactions. We thought that all these new findings were of relevance to the field and were worth publishing. However, to address the criticism focused on the lack of structures that show adhesive interfaces, we have obtained one additional structure (*hs* PCDH24 EC1-2 form II, PDB 7N86) with a crystal contact that we propose represents a bona fide *trans* interface for homophilic adhesion mediated by human PCDH24. Additional bead aggregation assays done with protein fragments carrying mutations inspired by this structure show altered adhesive capabilities that support the relevance of this interface for PCDH24 function. We have also used this *in-vitro* validated interface to propose a model for the PCDH24 / CDHR5 complex. While we acknowledge that the data presented does not fully resolve all the structural determinants of the homophilic and heterophilic PCDH24 and CDHR5 interactions, we strongly believe that our revised report, with the new data, does describe major advances in this field.

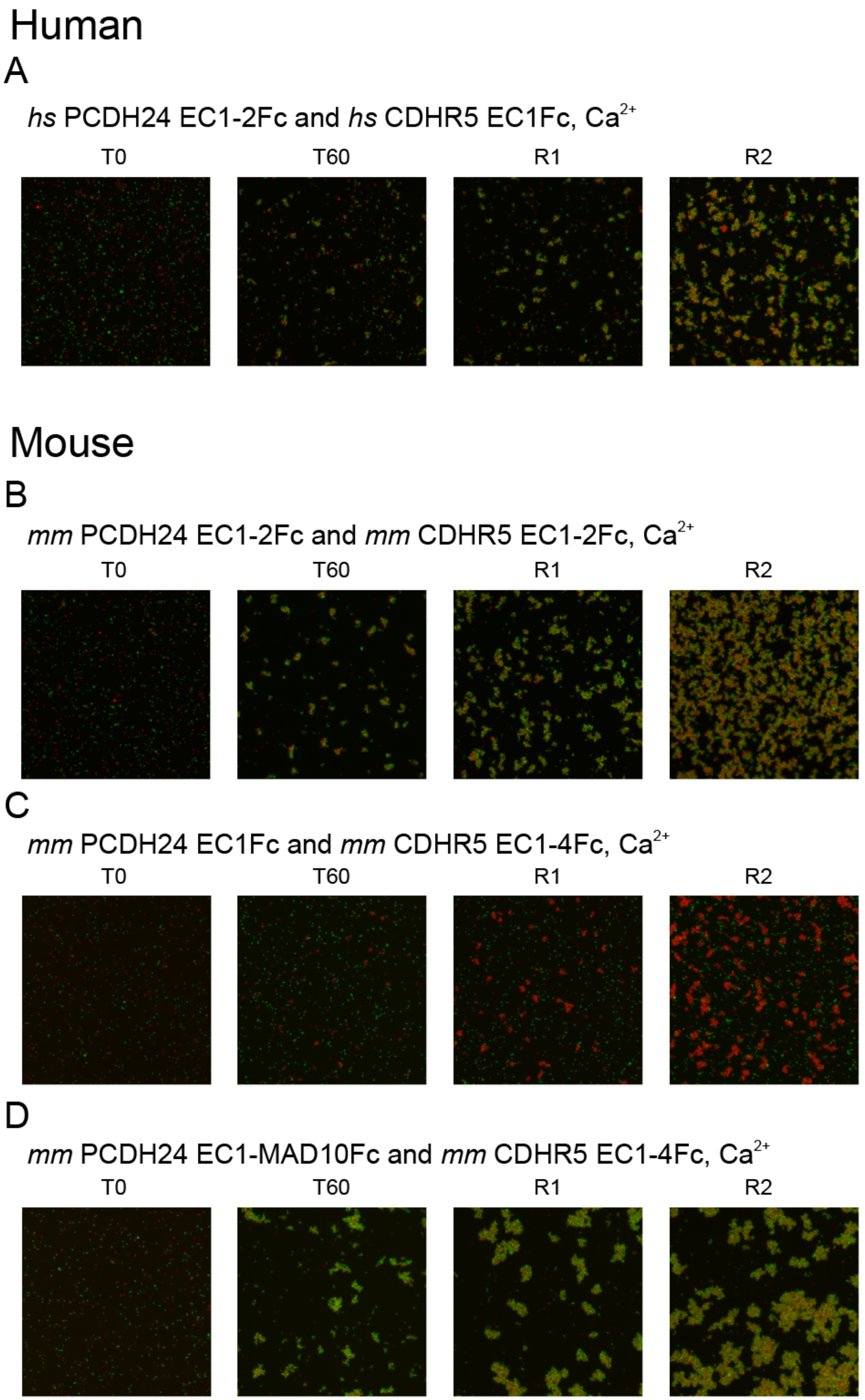
Human
A
hs PCDH24 EC1-2Fc and hs CDHR5 EC1Fc, Ca2+
T0
T60
R1
R2
Mouse
B
mm PCDH24 EC1-2Fc and mm CDHR5 EC1-2Fc, Ca2+
T0
T60
R1
R2
C
mm PCDH24 EC1Fc and mm CDHR5 EC1-4Fc, Ca2+
T0
T60
R1
R2
D
mm PCDH24 EC1-MAD10Fc and mm CDHR5 EC1-4Fc, Ca2+
T0
T60
R1
R2

Heterophilic binding assays at various time points. (A-D) Images from binding assays using the human minimum heterophilic adhesive units *hs* PCDH24 EC1-2Fc and *hs* CDHR5 EC1Fc (A), the mouse minimum heterophilic adhesive units *mm* PCDH24 EC1-2Fc and *mm* CDHR5 EC1-2Fc (B), the non-aggregating *mm* PCDH24 EC1Fc and *mm* CDHR5 EC1-4Fc (C), and the full-length cadherin ectodomains *mm* PCDH24 EC1-MAD10Fc and *mm* CDHR5 EC1-4Fc (D). Green fluorescent protein A beads are coated with Fc-tagged PCDH24 fragments while red fluorescent protein A beads are coated with Fc-tagged CDHR5 in all panels. Images show bead aggregation observed at the start of the experiment (T0) and after 60 min (T60) followed by rocking for 1 min (R1) and 2 min (R2), all in the presence of 2 mM $CaCl_2$. Small aggregates mediated by heterophilic complexes are observed at T60 in (A) and (B). These aggregates get larger with rocking. In contrast, (C) shows red aggregates only, mediated by *mm* CDHR5 EC1-4Fc, which appear at T60 and grow bigger while single beads coated with *mm* PCDH24 EC1Fc (green) are present but do not aggregate. Unlike the experiments shown in (B), the minimum homophilic unit for mouse CDHR5 (*mm* CDHR5 EC1-3Fc) is present but does not cause aggregation of red beads as seen in (C). Only aggregates mediated by heterophilic complexes are observed. These data suggest that homophilic *trans* adhesion is not required for heterophilic *trans* adhesion.
