# Supplementary material for: Heterophilic and homophilic cadherin interactions in intestinal intermicrovillar links are species dependent
Source: PLoS Biol. 2021 Dec 6;19(12):e3001463. doi: 10.1371/journal.pbio.3001463 (PMC8691648; doi:10.1371/journal.pbio.3001463)
Supplement: Attachment — Submitted filename: ResponseReviewersFINALV0.pdf [file pbio.3001463.s043.pdf]

We thank all three reviewers for their thorough and thoughtful comments.

A detailed response follows, including reviewer comments in *italics*.

Reviewers' comments

Rev. 1:
The authors fully addressed all of my concerns and I recommend the manuscript for publication in PLOS Biology.

Thank you.

Rev. 2:
The authors addressed all my concerns and comments in a satisfactory way. This revised manuscript is clearly presented and makes a strong case that homophilic and heterophilic interactions by PCDH24 and CDHR5 are species dependent involving distinct minimal adhesive units in different species.

Thank you.

Rev. 3:
The authors have put forth a thorough and scholarly effort to respond to essentially all of the reviewer's comments. The addition of a second structure of hs PCDH24 EC1-2 that potentially shows a trans interface and the additional binding assays significantly strengthen the paper. I think that the article, in its current format, warrants publication in PLOS Biology.

Thank you.
